# Individualized Closed-Loop Acoustic Stimulation Suggests an Alpha Phase Dependence of Sound Evoked and Induced Brain Activity Measured with EEG Recordings

**DOI:** 10.1523/ENEURO.0511-23.2024

**Published:** 2024-06-14

**Authors:** Tylor J. Harlow, Samantha M. Marquez, Scott Bressler, Heather L. Read

**Affiliations:** ^1^ Department of Psychological Sciences, University of Connecticut, Storrs, Connecticut 06269; ^2^ Brain-Computer Interface Core, University of Connecticut, Storrs, Connecticut 06269; ^3^Institute of Brain and Cognitive Science (IBACS), University of Connecticut, Storrs, Connecticut 06269; ^4^Elemind Technologies, Inc., Cambridge, Massachusetts 02139; ^5^ Department of Biomedical Engineering, University of Connecticut, Storrs, Connecticut 06269

**Keywords:** alpha, attention, auditory, closed-loop, gating inhibition, individualized

## Abstract

Following repetitive visual stimulation, post hoc phase analysis finds that visually evoked response magnitudes vary with the cortical alpha oscillation phase that temporally coincides with sensory stimulus. This approach has not successfully revealed an alpha phase dependence for auditory evoked or induced responses. Here, we test the feasibility of tracking alpha with scalp electroencephalogram (EEG) recordings and play sounds phase-locked to individualized alpha phases in real-time using a novel end-point corrected Hilbert transform (ecHT) algorithm implemented on a research device. Based on prior work, we hypothesize that sound-evoked and induced responses vary with the alpha phase at sound onset and the alpha phase that coincides with the early sound-evoked response potential (ERP) measured with EEG. Thus, we use each subject’s individualized alpha frequency (IAF) and individual auditory ERP latency to define target trough and peak alpha phases that allow an early component of the auditory ERP to align to the estimated poststimulus peak and trough phases, respectively. With this closed-loop and individualized approach, we find opposing alpha phase-dependent effects on the auditory ERP and alpha oscillations that follow stimulus onset. Trough and peak phase-locked sounds result in distinct evoked and induced post-stimulus alpha level and frequency modulations. Though additional studies are needed to localize the sources underlying these phase-dependent effects, these results suggest a general principle for alpha phase-dependence of sensory processing that includes the auditory system. Moreover, this study demonstrates the feasibility of using individualized neurophysiological indices to deliver automated, closed-loop, phase-locked auditory stimulation.

## Significance Statement

Healthy adult brains generate alpha oscillations, and individual subjects have different alpha oscillation frequencies, which impact how they dynamically process and attend to sensory information. Yet, little is known about the fine-scale temporal dynamics between sensory events and alpha phase and corresponding neuromodulation of auditory input processing. Here we use a novel closed-loop technology and individualized approach to play sounds at specific frontal alpha phases. We demonstrate novel alpha phase-dependent effects on auditory evoked responses, alpha levels, alpha phase coherence, and frequency. This individually tailored closed-loop approach has potential applications for research and health applications for a variety of neurological, developmental, and clinical disorders.

## Introduction

Cortical alpha oscillations are altered in multiple disorders including tinnitus and attentional deficit disorders (Weisz et al., 2011; Deiber et al., 2020), providing a strong rationale for developing targeted neuromodulation to explore and potentially improve the underlying physiology. High alpha oscillation power is considered essential for inhibiting cortical and behavioral responses to “distracting” sounds including weak, task-irrelevant or actively ignored auditory stimuli (Dubé et al., 2013; Wöstmann et al., 2019; Clements et al., 2022). However, alpha mediated inhibition is cyclic with trough and peak phases in cortical alpha field potentials coinciding with excitable and inhibited neural activity states, respectively (Haegens et al., 2015; Lakatos et al., 2016; Myers et al., 2022). Hence, it has been proposed that cyclic alpha oscillations also enhance synchronized information processing across brain areas during the “excitable” phase of alpha (Klimesch 2012; Bonnefond et al., 2017; Lombardi et al., 2023). Consistent with this physiology, opposite phase-dependent effects are observed on the auditory evoked response potential (ERP) amplitudes and behavioral auditory event detection rates when sounds are played during the trough versus peak alpha phases measured with scalp electroencephalography (EEG; Kruglikov and Schiff, 2003; Neuling et al., 2012). In the visual system, the visual ERP amplitude and poststimulus alpha power both vary with the post hoc reconstructed alpha phase aligning with sensory events (Mathewson et al., 2009; Dou et al., 2022). One theory proposes that this phase dependence comes about due to dynamic interactions between early feedforward sensory evoked potentials and alpha oscillation phase in brain areas that generate both signals (Alexander et al., 2020). Supporting this idea, when the early visual ERP overlaps with the poststimulus trough versus peak phases of alpha, visual detection rates are enhanced versus suppressed and the poststimulus alpha power is reduced versus increased, respectively (Alexander et al., 2020). It remains unclear whether a similar physiological principle applies to alpha phase-dependent effects of auditory stimulation.

Here, we employ a novel closed-loop approach to track alpha oscillations and play sounds phase locked to individualized target phases measured at frontal EEG locations. As reviewed previously, several studies fail to show alpha phase-dependent effects with “open-loop” rhythmic auditory stimulation protocols and post hoc phase analyses alone (VanRullen et al., 2014; Zoefel and VanRullen, 2017). In contrast, alpha phase dependence of the auditory ERP measured with EEG has been demonstrated by detecting a voltage threshold level for the trough alpha phase and probing following phases at fixed time intervals (Kruglikov and Schiff, 2003). Fixed time intervals are essentially open-loop and can be problematic given known variations in alpha frequency and phase across subjects and time (Notbohm et al., 2016; Hoffman et al., 2023). To overcome this limitation, we adopt a closed-loop stimulation approach using a novel endpoint-corrected Hilbert transform (ecHT) algorithm implemented on a research device. Our group and others have shown previously that this ecHT algorithm mitigates the Gibbs distortion errors in instantaneous signal readout associated with standard Hilbert transforms and acausal filtering (Schreglmann et al., 2021; Bressler et al., 2023). In addition, we have subjects actively ignore sounds and play phase-locked sounds at quasiperiodic slow rates to increase alpha levels in the time window preceding sound onsets (Cabral-Calderin and Henry, 2022). With this approach, we obtain high prestimulus alpha levels needed for phase-dependent and average sensory stimulus induced modulation of alpha and evoked responses (Iemi et al., 2019; Dou et al., 2022). To test phase dependence, we deliver sounds phase locked to alpha oscillations measured at frontal locations because prior work finds high magnitude frontal alpha oscillations during baseline conditions (Bahramisharif et al., 2013; Halgren et al., 2019; Alamia et al., 2020; Pang et al., 2020) and during auditory tasks requiring subjects to ignore sounds (Dubé et al., 2013; Wöstmann et al., 2019). Additionally, our group and others observe short latency auditory evoked responses at frontal cortical locations in primates including humans (Knight et al., 1999; Marrouch et al., 2020). Here, we target alpha phases that account for natural variations in individualized alpha frequency (IAF; Haegens et al., 2014; Zibrandtsen and Kjaer 2020) and auditory ERP latencies across subjects (Prakash et al., 2016). Post hoc analysis confirms our approach effectively plays sounds at targeted trough and peak alpha phases which in turn aligns the early auditory evoked P1 potential with the following peak and trough phases, respectively. With this approach, we observe alpha phase-dependent effects on the auditory ERP and alpha oscillations. These results support feasibility to use a novel closed-loop and individually tailored sensory stimulus approach for future exploration of bidirectional neuromodulation of auditory ERP potentials and poststimulus alpha oscillations.

## Materials and Methods

### Ethics statement and participants

All subjects in this study gave written informed consent for participation and to share their deidentified data with Elemind Technologies according to our protocol approved by the University of Connecticut Institutional Review Board. Subjects were compensated at an approved hourly rate for participating in the experiment. Data for three out of 22 subjects total was not included in the present study due to incomplete datasets across conditions or extensive signal artifacts. A total of nineteen young adults (9 females, 10 males) ages 18–32 [mean ± SD = 24.47 (±5.8)] participated in this study. All participants had normal or corrected-to-normal vision and intact hearing ability with clear ear canals and no history of neurological disorders and confirmed they were not taking medications. A subset of data presented here appears in a preprint report (Bressler et al., 2023) and the mean ERP-P1 latency from this subset was used in a separate study to set fixed alpha phases (Bressler et al., 2023).

### EEG recording task paradigms

**Figure 1.**
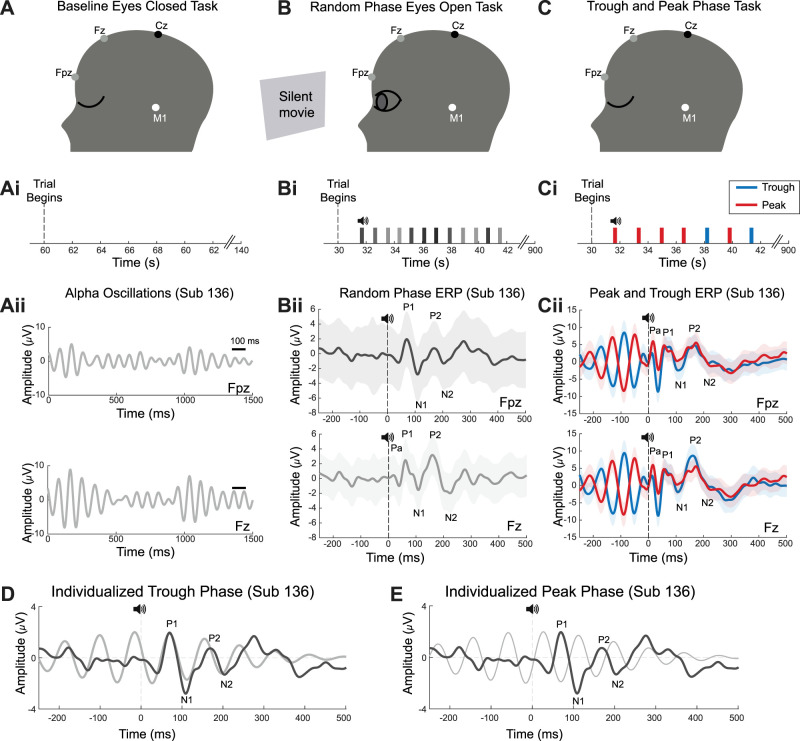
EEG recording and task conditions used to determine and test brain responses to individualized alpha phases. ***A–C***, Scalp EEG was recorded while subjects participated in three separate task paradigms. There were two recording locations including frontal (Fpz) and prefrontal (Fz) locations referenced to the left mastoid (M1). ***A****,* ***Ai***, The first task is the “baseline eyes closed” task where no sounds are played as EEG data was collected for a total duration of 2 min in all subjects. ***Aii***, EEG recording of alpha band activity in the same time window for frontal (Fpz) and prefrontal (Fz) recording locations in the example subject (Sub 136). ***B***, ***Bi***, For the “random phase eyes open task,” after a 30 s baseline, pink-noise sounds are delivered phase locked to random alpha phases with an average ISI of 927 ms for a total of 15 min (aka 900 s). ***Bii***, Average auditory ERP obtained at frontal (Fpz, top) and prefrontal (Fz, bottom) locations for an example subject (Sub 136). ***C***, ***Ci***, For the “trough and peak phase task,” after a 30 s baseline, pink-noise sounds are delivered phase locked to random alpha phases with an average ISI of 1,636 for a total of 15 min (aka 900 s). ***Ciii***, Average auditory ERP for peak and trough phase-locked sounds at frontal (Fpz, top) and prefrontal (Fz, bottom) locations. ***D***, ***E***, Schematic illustration of how the individualized alpha peak and trough phases are estimated for an example subject (Sub 136). Here and elsewhere, the solid line denotes the mean and the light shaded area the standard error of the mean (SEM).

**Figure 2.**
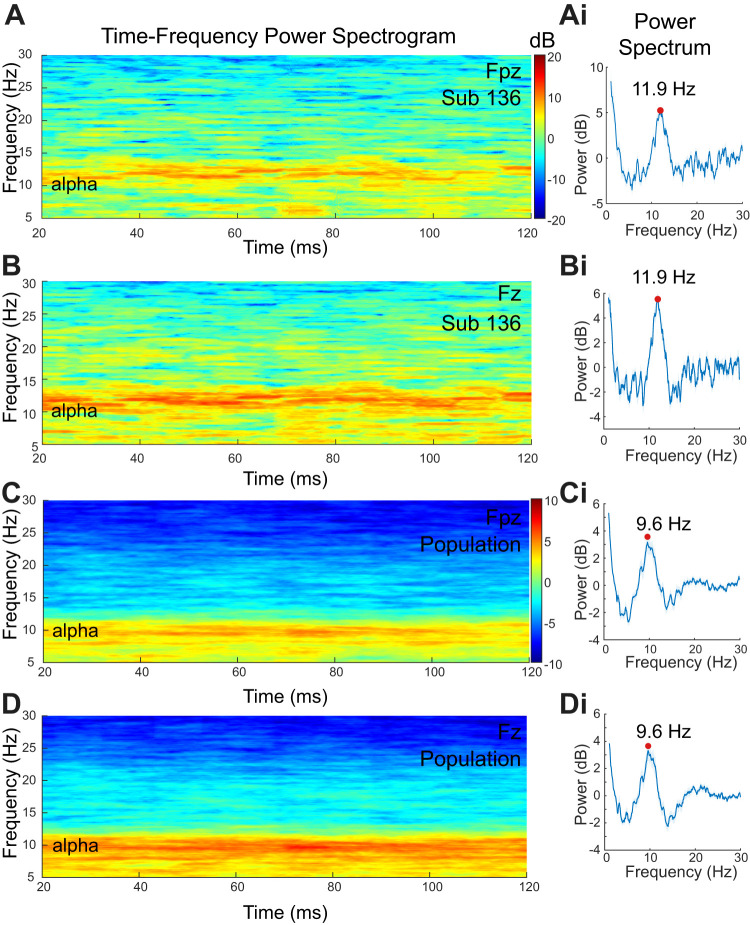
Estimation of IAF. ***A***, ***B***, Multitaper time-frequency power spectrograms for the example subject (Sub 136) measured at the frontal (***A***, Fpz) and prefrontal (***B***, Fz) locations. ***Ai***, ***Bi***, Detrended spectral density plots used to find the frequency with maximum power as an estimate of IAF for an example subject (Sub 136). Red dot indicates the location of the power maximum in each plot. ***C***, ***D***, The population average of multitaper time-frequency power spectrograms (*n* = 19) for the frontal (***C***, Fpz) and prefrontal (***D***, Fz) locations. ***Ci***, ***Di***, Corresponding population average detrended PSD plots for frontal (***Ci***) and prefrontal (***Di***) locations. As the power tends to be lower for people with higher frequency IAF, the population average spectrogram yields IAFs of 9.6 and 9.67 Hz at frontal (Fpz) and prefrontal (Fz) locations, whereas the average of individual IAFs is closer to 10 Hz (see text).

**Figure 3.**
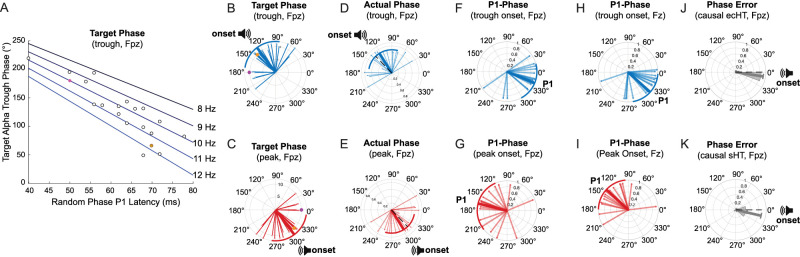
Individualized trough and peak sound onset phases align auditory evoked P1 potentials with poststimulus peak and trough phases with minimal phase-locking error. ***A***, As illustrated for trough phase, the target phases vary systematically with the ERP-P1 latency and IAF (lines Hz) across subjects (*n* = 19). ***B–H***, Radial plots of individual subject phases (thin lines, circles), population means (thick line), and standard deviations (circular arc). ***B***, ***C***, Radial plots show the opposing distributions of target trough and peak phases which have means (standard deviations) of 127° (44°) and 307° (44°), respectively. This corresponds to opposing (180° shifted) alpha phases. In ***A–C***, the orange filled circles denote the individualized phases for Subject 136 who has an IAF of 11.9 Hz (as in Figs. 1 and 2). In ***A–C***, the pink filled circles show a hypothetical subject with an IAF of 10 Hz and an ERP-P1 latency of 50 ms for reference. ***D***, ***E***, Radial plots show the distribution of actual trough and peak phases which have means (standard deviations) of 127 (−64) and 308 (−68.85) angular degrees, respectively. This corresponds to opposing (181° shifted) alpha phases. The target (***B***, ***C***) and actual (***D***, ***E***) phase-locked phases are not significantly different at the frontal (Fpz) location (Watson's *U*2 test: trough *U* = 0.002 and *p* = 0.350; peak *U* = 0.005, *p* = 0.300). ***F***, ***G***, At frontal (Fpz) locations, with frontal trough and peak phase-locked sounds, the auditory evoked P1 potentials occur at 337° (38°) and 166° (53°), respectively. Thus, the P1 potentials arrive in opposing (171° shifted) phases with trough and peak sound onsets at Fpz. ***H***, ***I***, At prefrontal (Fz) locations, with frontal trough and peak phase-locked sounds, the auditory evoked P1 potentials occur at 318° (39°) and 137° (42°), respectively. Thus, the P1 potentials arrive in opposing (181° shifted) phases with trough and peak sound onsets at Fz. ***J***, ***K***, The mean actual phases achieved with real-time ecHT phase-locking are close to the target phase. ***J***, The PLV is 0.92(0.02) and the phase-locking error relative to target phase is −9° (5) with the ecHT causal filter signal processing. ***K***, The mean PLV is 0.68 (0.12) and the PE is −13.04° (32) with standard Hilbert transform (sHT) causal filter signal processing. The length of the mean vector denotes the mean, with the arc corresponding to the standard deviation in PEs across participants. Radial histogram phase analysis and radial plots are based on a cosine function estimate of the alpha oscillations and using a bin size of 20°. Data include *n* = 19 subjects and additional pink dot is a reference point.

**Figure 4.**
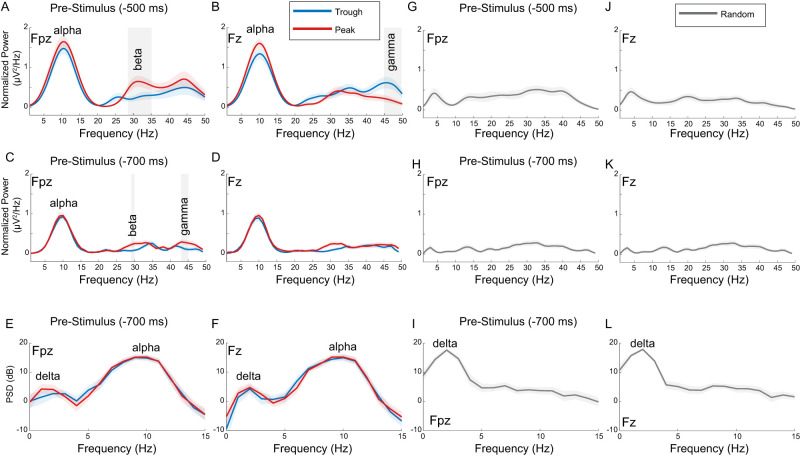
High prestimulus alpha levels with “trough and peak phase task” assure potential to phase lock to alpha. ***A–D***, EEG data obtained during the “trough and peak phase task” condition, yields high alpha levels at −500 ms (***A***, ***C***) versus −700 ms (***B***, ***D***) prior to sound stimulus onset at frontal (Fpz) and prefrontal (Fz) locations. Alpha levels are highest in the −500 ms time window before sound onsets (***A***, ***B***). Permutation testing with post hoc cluster analyses confirms that prestimulus alpha power does not vary with trough versus peak phase (*p* > 0.05). Beta is significantly greater for peak versus trough phases at the frontal (Fpz, ***A***, ***C***) location in the −500 and −700 ms prestimulus windows with *p* = 0.011 and *p* = 0.008, respectively. Gamma is significantly greater for peak versus trough at frontal (Fpz, ***C***) locations and greater for trough versus peak at prefrontal (Fz, ***B***) locations with *p* = 0.0101 and *p* = 0.020, respectively. ***E***, ***F***, Prestimulus alpha levels are also high with a second analysis that optimizes quantification of power spectral frequencies that are time locked and phase locked to sound onsets in the prestimulus time window (−700 ms) at both locations (Fpz, Fz). In addition, this approach finds high delta band power in the prestimulus time window likely due to the slow quasi periodic ISIs used for playing phase-locked sounds. Permutation testing finds no differences in delta or alpha power levels across phase-locked conditions. ***G–K***, Data obtained during the “random phase task” condition, standard non-normalized PSD analysis confirms that alpha power is at low levels at −500 ms (***G***, ***J***) and −700 ms (***H***, ***K***) prior to sound stimulus onset at frontal (Fpz) and prefrontal (Fz) locations. ***I***, ***L***, With the “random phase task” condition, alpha power also is low in the prestimulus time window (−700 ms) using the second method of analysis as in ***E*** and ***F*** panels.

**Figure 5.**
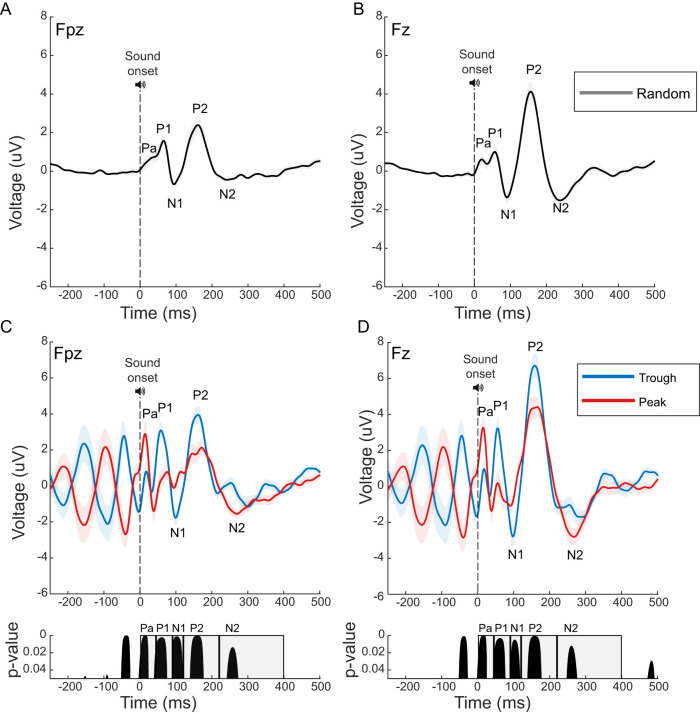
Amplitudes of the average auditory ERP components vary with individualized alpha phase, but prestimulus alpha levels do not. ***A***, ***B***, The pink-noise auditory ERP response acquired during the “random phase eyes open task” and averaged across all subjects (*n* = 19) for frontal (Fpz, ***A***) and prefrontal (Fz, ***B***) locations. ***C***, ***D***, Phase-dependent differences are observed across five components (Pa, P1, N1, P2, N2) of the subject averaged auditory ERP at frontal (Fpz, ***C***) and prefrontal (Fz, ***D***) locations. Pa and N2 potentials are smaller for the trough (blue) versus peak (red) phase condition. In contrast, P1, N1, and P2 components are all larger for the trough (blue) versus peak (red) condition. ***C***, ***D***, Bottom, Permutation tests followed by cluster analysis (Materials and Methods) confirms significant phase-dependent differences across all five ERP components. For frontal (Fpz, ***C***) location, the maximum *p* values for Pa, P1, N1, P2, and N2 are 0.0003, 0.0021, 0.0010, 0.0006, and 0.0134 at 16, 60, 102, 160, and 257 ms time points, respectively. For prefrontal (Fz, ***D***) location, the maximum *p* values for Pa, P1, N1, P2, and N2 are 0.0002, 0.0026, 0.0040, 0.0008, and 0.0101 at 16, 62, 104, 158, and 260 ms time points, respectively. Shaded boxed areas correspond to the temporal windows used to compare ERP components here and for data shown in Figure 6 used to run additional pairwise *t* tests (Table 1).

**Figure 6.**
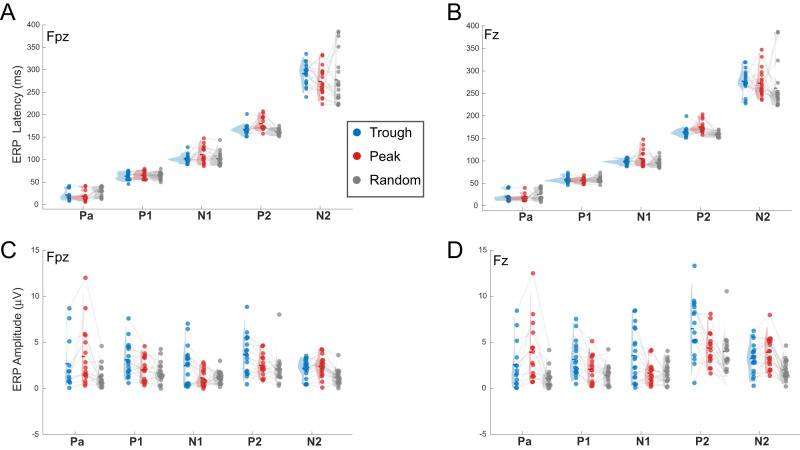
Auditory ERP component latencies are consistent across phase conditions but amplitudes vary with peak versus trough. ***A***, ***B***, No significant differences are observed for ERP component latencies (Pa, P1, N1, P2, and N2) for trough (blue), peak (red), and random (gray) phase conditions at frontal (***A***, Fpz) or prefrontal (***B***, Fz) locations. Multiway ANOVA of ERP latency finds significant differences across electrodes (*F*_(1,144)_ = 10.08; *p* = 0.002), but no main effect of condition (*F*_(2,72)_ = 2.05; *p* = 0.130). ***C***, ***D***, Multiway ANOVA of ERP amplitude finds significant differences across electrodes [Fpz vs Fz; (*F*_(1,144)_ = 22.88; *p* = 0.0000023) and trough, peak, and random phase conditions (*F*_(2,72)_ = 27.86; *p* = 0.0000000000033). Notably, the early Pa component is smaller amplitude for trough versus peak conditions, whereas the P1 and N1 components are larger for trough versus peak phase condition. See Table 1 for full statistical comparisons of trough versus peak phase condition effects on latency and amplitude. For above figures, means (horizontal lines), individual values (colored dots), and distribution (half-violin) plots are shown for each measure. Light gray lines show within subject measures across phase conditions.

EEG recording paradigms were designed to test the hypothesis that sound played phase locked to frontal (Fpz) individually tailored trough or peak alpha phases have distinct effects on auditory ERP and alpha oscillations. Primate frontal and auditory cortices both have short latency auditory evoked responses, as demonstrated with intracranial recordings by our group (Marrouch et al., 2020) and others (Howard et al., 2000; Happel et al., 2010; Haegens et al., 2015; Komatsu et al., 2015; Kajikawa et al., 2017). Moreover, frontal and auditory cortices are known to generate alpha oscillations under a variety task conditions (Haegens et al., 2015; de Pesters et al., 2016; Lakatos et al., 2016; Bonnefond et al., 2017; Billig et al., 2019). Robust frontal alpha oscillations are observed with eyes closed and localized to superficial radial dipole layers with more robust signals measured with EEG, as compared with MEG (Srinivasan et al., 2006). Thus, we tested feasibility to play sounds phase locked to alpha measured with frontal (Fpz) EEG electrodes, as illustrated (Fig. 1). In addition, we recorded from a prefrontal (Fz) location and referenced both electrodes to the left mastoid (M1). All EEG recordings were carried out inside a sound isolation room while subjects participated in three task paradigms (Fig. 1). For the “baseline eyes closed task,” participants closed their eyes and sat quietly while EEG was recorded for an average of 2.36 (0.32 s) following a 60 s baseline in order to quantify their IAF, as detailed below (Figs. 1*A*, 2*A*). In the second “random phase eyes open task,” participants watched a silent cartoon video in a relaxed sitting position and were instructed to ignore sounds (Fig. 1*B*), as in prior auditory evoked response studies (Choi et al., 2013; Prakash et al., 2016). Prior studies found alpha levels are reduced with eyes open which is hypothesized to be due to increased allocation of attention to auditory sensory input (Clements et al., 2022). Likewise, here the “random phase eyes open task” resulted in reduced prestimulus alpha power levels which allowed us to measure the auditory ERP component latencies independent of ongoing alpha (detailed below). Data from these first two tasks was used to estimate the individually tailored alpha trough and peak phases aligning the sound evoked positive, P1, response to the following peak and trough phases, respectively (Figs. 1*D*,*E*, 3). The third, “trough and peak phase task,” was designed to keep subjects alert, while ignoring sounds with the goal of promoting high prestimulus alpha power levels. This task consisted of two back-to-back 15 min sessions where participants were instructed to ignore auditory stimuli while they carried out “mental arithmetic” (Palva et al., 2005), while sitting in a relaxed position with eyes closed. The “mental arithmetic” was to rehearse a newly assigned 11 by 1 (double digit) multiplication table on which they were quizzed following the session. For example, the number 13 multiplied by each number in the series spanning from 10 to 21. Following each 15 min session, subjects verbally reported the 11 multiplications and no quantitative analysis was performed. Post hoc alpha power analyses detailed below confirmed that prestimulus alpha power was high in the “trough and peak phase task” used to quantify phase-dependent differences (Fig. 4). The auditory ERP latencies and amplitudes were compared across random, trough, and peak phases, as detailed below (Figs. 5, 6; Table 1).

**Table 1.** Auditory ERP component latencies (top rows) and amplitudes (bottom rows) across individualized trough, peak, and random phase-locked sound conditions for both electrodes

Phase variations in latency (*t* test, post hoc correction)					
ERP component	Electrode	Random phase (ms)	Trough phase (ms)	Peak phase (ms)	Peak versus trough (*p* value)
Pa	Fpz	28.69 (10.73)	21.29 (11.59)	18.59 (10.40)	0.9979
P1	Fpz	64.32 (7.55)	63 (8.33)	65.40 (8.06)	0.9979
N1	Fpz	102.22 (14.28)	102 (8.97)	111.19 (18.25)	0.1794
P2	Fpz	161.93 (6.64)	167 (10.92)	180.01 (14.49)	0.1028
N2	Fpz	277.66 (56.47)	291 (23.99)	273.98 (31.18)	0.1521
Pa	Fz	25.24 (12.46)	20.81 (11.17)	17.96 (7.39)	1.0000
P1	Fz	58.55 (6.73)	58.21 (7.02)	56.55 (5.42)	0.9979
N1	Fz	95.46 (9.13)	98.02 (5.68)	105.17 (18.01)	0.2519
P2	Fz	158.18 (5.57)	164.26 (10.84)	175.27 (12.80)	0.1521
N2	Fz	261.27 (49.63)	277.03 (25.64)	272.93 (30.60)	0.9979
Phase variations in amplitude (*t* test, post hoc correction)					
ERP component	Electrode	Random phase (uV)	Trough phase (uV)	Peak phase (uV)	Peak versus trough (*p* value)
Pa	Fpz	1.52 (1.69)	2.57 (3.01)	3.49 (3.27)	0.0355
P1	Fpz	1.96 (1.61)	3.08 (1.96)	1.20 (2.16)	0.0355
N1	Fpz	−0.90 (1.01)	−2.44 (2.14)	−1.02 (1.06)	0.0261
P2	Fpz	2.44 (2.23)	3.67 (2.21)	2.53 (1.18)	0.1171
N2	Fpz	−0.94 (0.58)	−2.19 (0.97)	−2.42 (1.08)	0.1853
Pa	Fz	1.20 (1.22)	1.98 (3.08)	3.92 (3.33)	0.0261
P1	Fz	1.45 (1.43)	2.95 (2.23)	0.71 (2.5)	0.0355
N1	Fz	−1.71 (1.22)	−3.56 (2.81)	−1.49 (1.39)	0.0355
P2	Fz	4.23 (2.23)	6.48 (3.15)	4.38 (1.87)	0.1171
N2	Fz	−1.76 (0.93)	−3.25 (1.61)	−3.91 (1.67)	0.0355

Latencies and amplitudes here are extracted from fixed time windows, and a pairwise *t* test compares peak versus trough phase conditions with post hoc Benjamini–Hochberg correction. Standard deviation given in parentheses.

### Sound design

All EEG recording and sound presentations were carried out inside an electrically shielded, sound-attenuating chamber while participants sat upright on a firm padded chair with their head against a head rest. Based on prior work, sounds were designed to be short duration with a high maximal sound level in order to evoke large and short latency P1 and N1 components of the auditory ERP response and to be challenging to ignore (Picton et al., 1974; Schadow et al., 2007; Thaerig et al., 2008; Prakash et al., 2016; Fujita et al., 2022). Binaurally presented sounds were short duration (12 ms), loud (85 dB maximum sound level in both ears), broad spectral band pink-noise sound pulses. Pink-noise sound pulses were shaped with a basis-spline function to create smooth onsets and offsets with minimal spectral splatter artifacts, as detailed in our prior report (Lee et al., 2016). In the “random phase eyes open task,” sound pulses were delivered at randomly ordered alpha phases separated by an interstimulus interval (ISI) which ranged from 878 to 1,634 ms (1 ms increments) with a mean ISI of 0.927 s (0.171 s) corresponding to a stimulus rate of ∼1 Hz (Fig. 1*B*,*Bi*). In the “trough and peak phase task,” ISI ranged from 1,628 to 1,650 ms (10 ms increments) with a mean of 1.636 s (0.055 s) corresponding to a stimulus rate of 0.611 Hz (Fig. 1*C*,*Ci*). In both tasks, the auditory stimulus interval was designed to minimize adaptation and variation in amplitudes and latencies of the P1 component (Dolu et al., 2001). A Wald–Wolfowitz test for random sequences with Benjamini–Hochberg post hoc correction for multiple comparisons confirmed that sounds were delivered with randomly interleaved phases in both tasks (Magel and Wibowo, 1997). Pink-noise sounds were delivered through calibrated Etymotic earphones (ER4 microPro) with disposable clean tips. Timing of sound delivery was controlled by a custom research-grade brain–computer interface device by Elemind Technologies which recorded EEG to track instantaneous target alpha phase and deliver phase-locked sounds.

### Closed-loop phase-locking approach

For closed-loop phase-locking to alpha oscillations in the EEG, we used a research device (Elemind Technologies) that implements a novel ecHT algorithm to estimate the instantaneous alpha phase and play sounds phase locked in real time. In post hoc analysis, when a complete time course of EEG data is available, prior studies typically have used the standard Hilbert transform with acausal filtering. However, the standard Hilbert transform generates Gibbs distortion errors when reading out instantaneous phase in real time, as detailed previously (Schreglmann et al., 2021). These Gibbs distortion errors have been attributed to nonuniform convergence of the discrete Fourier transform (DFT) at the discontinuity between the beginning and endpoints of the calculated analytic signal. In contrast, the ecHT algorithm implements a causal bandpass filter centered around the frequency band of interest leading to a reduced contribution of off-frequency DFT coefficients. Prior studies have demonstrated that the ecHT algorithm mitigates the endpoint Gibbs distortion errors and allows for the high quality estimation of sample-by-sample oscillatory phase and amplitude in real time (Schreglmann et al., 2021). Data analyses and simulations implementing the ecHT algorithm found the Gibbs distortion errors were minimized provided the parameters of the bandpass filter were properly tuned to match the narrowband signal of interest (Schreglmann et al., 2021; Bressler et al., 2023). For detailed descriptions of how the ecHT algorithm works, please refer to this prior work (Schreglmann et al., 2021; Bressler et al., 2023).

### Data acquisition

Following scalp measurements to determine 10/20 EEG electrode locations, adhesive disposable Ag/Ag-Cl electrodes (Vermed) were placed at scalp locations corresponding to Fpz and Fz for positive leads and left mastoid for reference (Fig. 1). A ground electrode was placed at the left frontal position (F1). Unprocessed “raw” EEG signals were collected via the Elemind Technologies “ecHT” research device which tracked instantaneous alpha phase in real time and delivered pink-noise sound pulses at target alpha phases. The Fourier and ecHT signal processing and phase-locking that were implemented here through an ecHT research device were detailed previously (Schreglmann et al., 2021). Experimental input parameters including the IAF and target trough and peak phases, pink-noise sound pulse duration, decibel level, jittered stimulus intervals, baseline and total recording durations were set using LabVIEW scripts (National Instruments) and loaded on the device through a Dell computer interface.

### EEG preprocessing

All analyses were conducted in MATLAB (version 2023b). Additionally, the Chronux, Signal Processing, and Circular Statistics toolboxes (versions 2.12, 9.2, and 1.21, respectively) were used. Prior to all analyses, an acausal bandpass filter from 0.2 to 70 Hz was applied to the EEG which was recorded with a sampling frequency of 501 Hz. Data were epoched from −0.25 to 0.5 s relative to the onset of the stimulus presentation for presentation of evoked response oscillations (EROs), but from −0.25 to 0.9 s for broad- and narrowband power analyses in all tasks including sounds. Trials with gross artifact magnitudes squared exceeding 100 µV^2^ such as eyeblinks were removed.

### Spectral time-frequency analyses

For time-frequency signal analyses, we used Fourier decomposition with Hilbert or wavelet transforms which can generate comparable time and frequency resolution (Bruns, 2004). For IAF estimates detailed below, signals were processed with the multitaper Hilbert transform to generate time-frequency spectrograms (Fig. 2), which optimize spectral frequency resolution (Prerau et al., 2017). Multitaper spectrograms were generated using a Fourier transform and 4 temporal filters (tapers) with a 6 s sliding analysis window and step size of 150 ms (Prerau et al., 2017). To calculate post hoc phase-locking value (PLV) and phase error (PE; detailed below), we filtered individual trial data with a bandpass causal filter centered around IAF (±25%) followed by an ecHT transform implemented on the device (Fig. 3*J*). For comparison, the PLV and PE also were calculated using a standard Hilbert transform applied to the recorded data filtered with the same bandpass causal filter (Fig. 3*K*). To compute the instantaneous alpha frequency, we used the same bandpass causal filter centered at IAF followed by the endpoint corrected or standard Hilbert transform (detailed below). All remaining spectral time-frequency analyses were carried out using the Morlet wavelet transformation which enables high resolution in both frequency and time domains (Cohen 2019). Stimulus-related changes in frequency and power over time using the Morlet wavelet transform to generate broadband time-frequency power spectrograms (Figs. 8, 9) and alpha band time-power plots (Fig. 10). The Morlet wavelet transform was used for spectral decomposition as follows:

(1) $$\varphi =e^{-i2\pi ft}\cdot e^{\left((-t^{2})/{\left(2\sigma \right)}^{2}\right)}.$$

Here, “*i*” represents the complex variable, “*f*” is the center frequency, and “*σ*” *is* the time bandwidth of the wavelet. Wavelets were produced using 140 linearly spaced center frequencies *f* ranging from 1 to 70 Hz. Cycles (*c*) were linearly spaced from 5 to 12 with each center frequency, with the time bandwidth *σ* being the following:

(2) $$\sigma =\;\frac{c}{2\pi f}.$$

Signal envelopes were extracted by taking the absolute value of the wavelet transformed signals, whereas power was considered the magnitude squared. Phase values in radians were extracted using the MATLAB function called “*angle*”.

### IAF estimates

To estimate phases for delivering phase-locked sounds, we estimated each subjects IAF which is known to vary across individual adult subjects IAF (Haegens et al., 2014; Zibrandtsen and Kjaer 2020). The 1/*f* aperiodic component of the corresponding power spectral density (PSD) plot was fitted with a third-order polynomial and subtracted to determine the frequency with maximum power or IAF (Fig. 2). The MATLAB “*findpeaks*” function was used to the frequency with maximum power or IAF after specifying a range of 7.5–16 Hz (Fig. 2, dots).

### Calculating individualized target phase

Based on the physiology, we hypothesized that playing sounds at the trough or peak alpha phases which account for individual variations in alpha frequency and P1 latency would have distinct effects on auditory evoked brain responses (Fig. 1*D*,*E*). The auditory P1 potential latencies are known to vary across subjects (Prakash et al., 2016); hence, our target phases accounted for individual differences in P1 latency, as in prior studies in the visual system (Alexander et al., 2020). After measuring each subject's IAF (Fig. 2), we simulated the pre- and poststimulus alpha oscillations based on actual prestimulus alpha (Fig. 1*D*,*E*). To estimate the individualized trough onset phase, the P1_latency_ was multiplied by the IAF and converted to degrees as shown in Equation 3. The individualized peak phase was calculated with the same equation. All phase analysis and radial plots were based on a cosine function.

(3) $$\mathrm{Onset}{∠}^{\circ }\;=\;-360^{\circ }\;\times \;P1_{\mathrm{latency}}\times \mathrm{IAF}.$$

### Quantifying the alpha phase stationarity in prestimulus and poststimulus time window

As detailed above, we estimated target trough and peak phases for playing phase-locked sounds with the goal of optimizing the temporal overlap between the early auditory evoked P1 potential and the following peak and trough alpha phases, respectively. This assumes that alpha phase will remain unperturbed and stationary in the half-cycle preceding and following the phase-locked sound onset. To quantify this potential stationarity of alpha half-cycle phase, we ran a phase correlation analysis. A causal ecHT filtered EEG signal was used to quantify each individual subjects' alpha phase, as causal filters produce fewer temporal distortions for estimating prestimulus and poststimulus alpha phase (Ronconi et al., 2017). Circular correlations were estimated using the *circ_corrcc* function from the Circular Statistics Toolbox in MATLAB. Circular correlations were first Fisher *z*-transformed, and the absolute value was taken such that correlations ranged from [0 1]. With this approach, we find the alpha phase cycle preceding and following phase-locked sound onset was highly (*r* = 0.99 or 99%) correlated for trough and peak phase-locked sounds, supporting the idea that alpha phase remained stationary in the early time window preceding and following sound onset (see Results). As detailed below, we observed changes (nonstationarity) of alpha oscillation amplitude and frequency in later time windows following onset of the P1 potential and the first poststimulus half-cycle of alpha.

### Quantifying P1-alpha phase alignment

One goal of this study was to determine whether the auditory P1 evoked potential was temporally aligned to the alpha half-phase following the phase-locked sound onset. To address this, the P1 latency was measured with standard procedures, as detailed below. As in the above analysis, we estimated each individual subjects' prestimulus and poststimulus alpha phases using a causal ecHT transform which yielded low PE (Fig. 3*J*). With this approach, we confirm the actual (Fig. 2*D*,*E*) trough and peak phases for playing phase-locked sounds were close to the target phases (Fig. 3*B*,*C*). Accordingly, sound onsets for trough and peak phases preceded the true trough (180°) and peak (360°/0°) phases by 53° and 52°, respectively. Next, for each individual subject, we determined the poststimulus alpha phase corresponding to the time of their individual P1 potential latency. We confirmed that trough and peak phase-locked sounds evoked an average P1 latency that preceded the true maximum peak (360°/0°) and trough (180°) phases by 23° and 14°, respectively (Fig. 4*F*,*G*, thick blue line). Thus, frontal alpha trough and peak phase-locked sounds generated P1 potentials that preceded the following individualized peak and trough phases by 18° (∼5 ms) on average. When sounds were played phase locked to frontal (Fpz) trough and peak phases, the P1 potential at the prefrontal location (Fz) preceded the true maximum peak and trough phases by 42° and 43° (∼12 ms), respectively, on average (Fig. 4*H*,*I*). Using a Watsons *U*2 test along with the causal ecHT signal filtering, we found this P1-phase alignment was not significantly different between electrodes (trough *U*2 = 0.384, *p* = 0.084; peak *U*2 = 0.368, *p* = 0.086). Thus, even though our closed-loop phase-locking targeted the more frontal (Fpz) location, a similar P1-alpha phase relationship was observed at the prefrontal location (Fz). For comparison, the P1-phase alignment also was determined for signals filtered with an acausal standard Hilbert Transform. With acausal filtering of frontal (Fpz) signals, the trough and peak phase-locked sounds were delivered at 126° (55°) and 308° (44°), respectively. With acausal filtering, for trough and peak phase-locked sounds, the P1 latencies on average coincided with poststimulus phases of 356° (39°) and 33° (75°), respectively. This inconsistent P1-phase alignment for signals filtered with an acausal standard Hilbert transform presumably reflects the temporal integration across prestimulus and poststimulus time windows on the order of 200 ms with acausal filters, as shown previously (Ronconi et al., 2017).

### PLV and PE analysis

The PLV was computed for each subject and averaged to estimate the trial-by-trial probability that the actual phase was the same as the target phase (Fig. 3*J*,*K*). The PLV corresponds to the absolute value of the mean phase difference $\left(\Delta \theta_{i}(t)\right)$ between target and actual phase at time (*t*) expressed as a complex phase vector (Eq. 4; *n* = number of trials), as detailed previously (Lachaux et al., 1999; Aydore et al., 2013). The PLV ranges from 0 to 1, with a value of 1 indicating a 100% correspondence between target and actual phase across all stimulus trials. Additionally, the PE was computed to estimate the mean difference between actual versus target phase in degrees angle (Fig. 3*J*,*K*). The PE therefore indexes how far off the target phase-locking was on average. To compute PLV and PE, data was preprocessed using the endpoint corrected (ecHT) or a standard Hilbert (sHT) transform coupled with a causal filter. For the real-time automated phase calculation, the ecHT was coupled with an alpha band delimited causal filter that requires no information about the signal in the future from the time point the phase is calculated. For comparison, we use a standard Hilbert transform with causal filters. In all cases, the filters were band delimited based on each subject's IAF with the same narrowband delimitation (±25%) used during real-time phase tracking. Group statistics are obtained by evaluating the mean and standard error across participants.

(4) $$\mathrm{PLV}=\left|\;\frac{1}{N}\overset{N}{\underset{n=1}{\sum }}\;e^{i\Delta \theta_{i}(t)}\right|.$$

### ERP analyses

To determine if there were alpha phase-dependent effects on the auditory ERP, we measured and compared auditory ERP amplitudes and latencies across task conditions (Figs. 5, 6; Table 1). For each subject, the auditory ERP was generated by smoothing the EEG signal with a fourth-order bandpass filter within the broad frequency range of 2–34 Hz, followed by averaging across trials. Smoothing using the bandpass filter was utilized for both visualization and subsequent analyses. ERP components were extracted using the MATLAB “*findpeaks*” function to locate maxima within time windows corresponding to each component. Consistent with prior work, auditory ERP component amplitudes and latencies were measured within the following time windows (Fig. 5, Table 1): Pa (1–45 ms), P1 (45–80 ms), N1 (80–150 ms), P2 (150–220 ms), and N2 (220–400 ms; Knight et al., 1999; Kisley et al., 2001; Eggermont and Ponton, 2002; Prakash et al., 2016). Grand average ERPs correspond to the arithmetic mean across subjects (Figs. 5, 6). As reported, we found minimal (<2 ms) and insignificant individual subject differences in the mean P1 latencies during the Random Phase Task versus the trough (mean difference = 1.11 ± 8.42 ms; *t* test *p* = 0.584) or peak (mean difference = 0.50 ± 11.56 ms; *t* test *p* = 0.865) phase-locked conditions at frontal (Fpz) locations. This mean difference across individual subjects was similar to the population mean differences which were statistically insignificant, as shown in Table 1. Thus, the P1 latency appears to be consistent across task conditions when examined on an individual basis and across the population. In the Results section, we report how auditory ERP component amplitudes vary with individualized trough and peak alpha phase at sound onset.

### EROs

**Figure 7.**
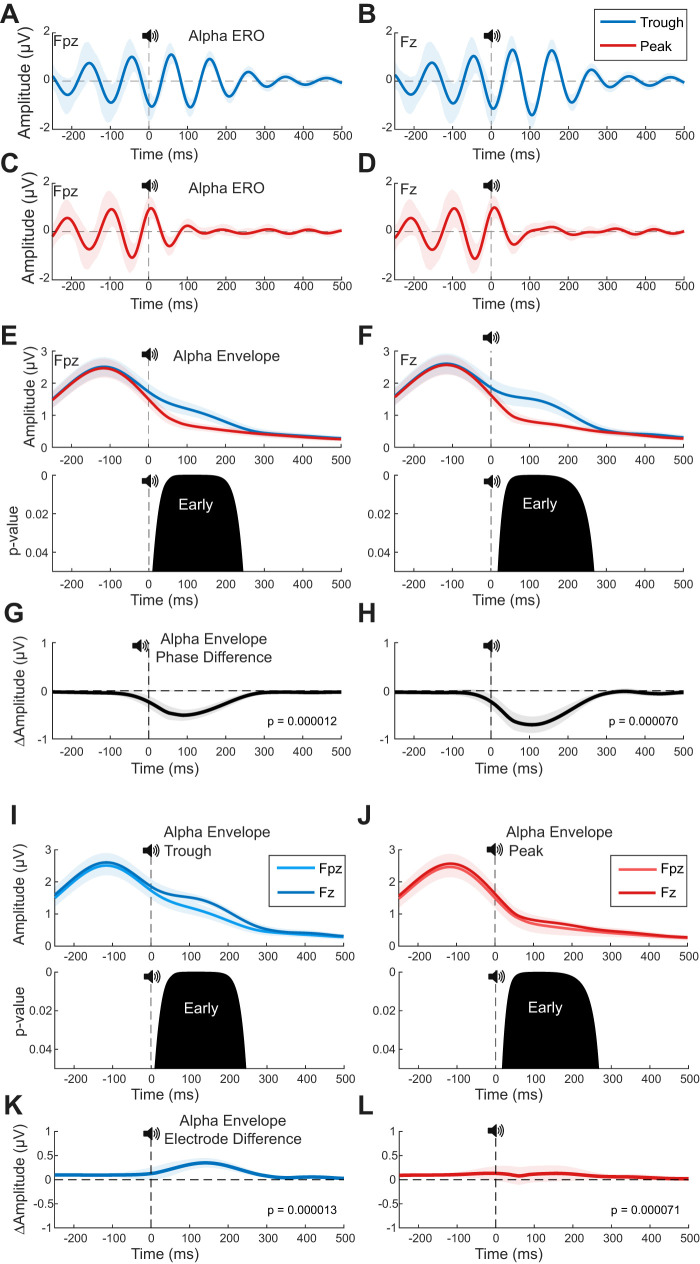
Phase dependence and electrode differences for the alpha auditory ERO. ***A–D***, The alpha band delimited ERO varies across trough (***A***, ***B***, blue lines) and peak (***C***, ***D***, red lines) phase conditions at frontal (***A***, ***C***, Fpz) and prefrontal (***B***, ***D***, Fz) locations. ***A***, ***B***, For trough phase condition, alpha oscillations remain high magnitude following sound onset (*t* = 0) at both locations. ***C***, ***D***, For peak phase condition, alpha oscillations are reduced following sound onset at both locations. ***E***, ***F***, The population average alpha ERO envelope is significantly higher for trough versus peak phase conditions at both locations. For Morlet wavelet transformed data shown here, permutation tests find significant differences in the early time window (50–250 ms) at both locations. For frontal (***E***, Fpz) and prefrontal (***F***, Fz) locations, the maximum difference is observed at 92 ms (*p* = 0052) and at 111 ms (*p* = 0.0087), respectively (bottom, *p* value distribution). Similar results are observed with a standard Hilbert transform (data not shown) with a *p* < 0.004, for the 50–250 ms time window at both locations (Methods). ***G***, ***H***, The phase-dependent difference (trough minus peak) is larger for the prefrontal (***H***, Fz) versus frontal (***G***, Fpz) locations. ***I***, ***J***, ERO envelopes are larger for the prefrontal location for both trough (***I***, Fz, dark blue line) and peak (***J***, Fz, dark red line) phase conditions. Permutation tests find differences by location are significant in the early time window before 250 ms (bottom, *p* value distribution). All plots here use the Morlet wavelet transform for spectral time-frequency analysis. Dark lines correspond to the means and light shaded areas the standard errors. The same phase-dependent effects were similarly significant (*p* < 0.001, for 50–250 ms) over the early poststimulation time window when analyzed with a standard Hilbert transform.

EROs were quantified using the same basic approach used for analyzing the auditory ERPs; however, the Morlet wavelet transformation was performed to isolate specific frequency ranges of interest. For example, alpha EROs were calculated by taking the average across trials for each participant at the wavelet corresponding to that subject's measured IAF, with the grand average alpha ERO being the mean across participants (Fig. 7).

### Stimulus evoked and induced effects

**Figure 8.**
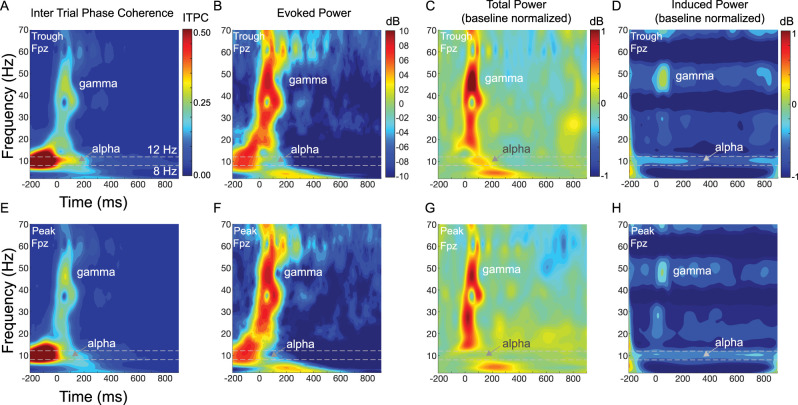
Broadband time-frequency plots illustrate phase-dependent differences in evoked and induced responses at the frontal (Fpz) location. ***A***, ***B***, ***E***, ***F***, Alpha ITPC and evoked power in the alpha range (dotted lines, 8–12 Hz) changes poststimulation (time = 0) in a phase-dependent manner with trough (***A***, ***B***) versus peak (***E***, ***F***) phase conditions. Independent of alpha phase, there is a time delayed peak in lower gamma frequency (35–60 Hz) range that coincides with the auditory ERP and a drop in alpha power relative to prestimulus baseline (0 to −200 ms). ***A***, ***B***, With trough phase condition, alpha power remains elevated within a narrow frequency band (arrow, alpha) poststimulation (arrow, alpha). ***E***, ***F***, With peak phase condition, alpha power and frequency (arrow, alpha) both decrease more so than with the trough phase condition. ***C***, ***G***, Baseline normalized Total Power also changes in a phase-dependent manner following sound onset. ***C***, For trough phase, total power in the alpha range (dotted lines) increases (red, positive dB) and then decreases (green-blue, negative dB) in the early (0–300 ms) and late (>300 ms) time windows following sound onset, respectively. ***G***, For peak phase, total power in the alpha range decreases (green-blue) and increases (yellow-green) in the early (0–300 ms) and late (>300 ms) time windows, respectively. ***D***, ***H***, Phase-dependent differences in the baseline normalized induced power are evident as a larger alpha desynchronization (aka power decrease, dark blue) in the late (>300 ms) time window for trough (***D***) versus peak (***H***) phase conditions.

**Figure 9.**
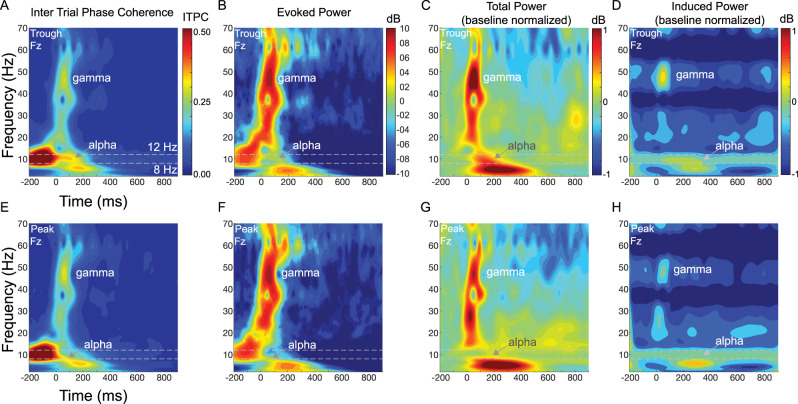
Broadband time-frequency plots illustrate phase-dependent differences in evoked and induced responses at the prefrontal (Fz) location. ***A***, ***B***, ***E***, ***F***, Alpha ITPC and evoked power in the alpha range (dotted lines, 8–12 Hz) changes poststimulation (time = 0) in a phase-dependent manner with trough (***A***, ***B***) versus peak (***E***, ***F***) phase conditions. Independent of alpha phase, there is a time delayed peak in lower gamma frequency (35–60 Hz) range that coincides with the auditory ERP and a drop in alpha power relative to prestimulus baseline (0 to −200 ms). ***A***, ***B***, With trough phase condition, alpha power remains elevated within a narrow frequency band (arrow, alpha) poststimulation (arrow, alpha). ***E***, ***F***, With peak phase condition, alpha power and frequency (arrow, alpha) both decrease more so than with the trough phase condition. ***C***, ***G***, Baseline normalized total power also changes in a phase-dependent manner following sound onset. ***C***, For trough phase, total power in the alpha range (dotted lines) increases (red, positive dB) and then decreases (yellow-green-blue, negative dB) in the early (0 to 300 ms) and late (>300 ms) time windows following sound onset, respectively. ***G***, For peak phase, total power in the alpha range decreases (green-blue) and then increases (yellow) in the early (0–300 ms) and late (>300 ms) time windows, respectively. ***D***, ***H***, Phase-dependent differences in the baseline normalized induced power are evident as a larger alpha desynchronization (aka power decrease, dark blue) in the late (>300 ms) time window for trough (***D***) versus peak (***H***) phase conditions.

**Figure 10.**
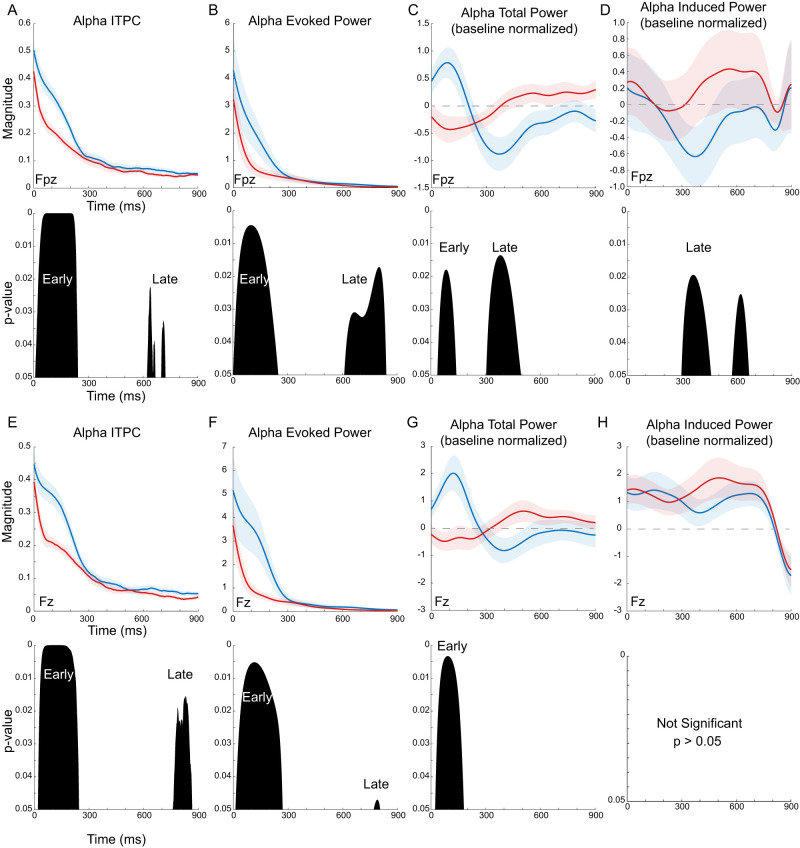
Alpha band delimited evoked and induced responses vary distinctly across individualized trough and peak alpha phase conditions. ***A***, ***B***, ***E***, ***F***, The evoked alpha phase coherence (ITPC, ***A***, ***E***) and evoked alpha power (***B***, ***F***) are both markedly higher for the individualized trough (blue lines) versus peak (red lines) phase conditions in the early (0 to 300 ms) time window at both locations (Fpz, Fz). ***A***, Bottom, Permutation testing with post hoc cluster analyses finds evoked alpha phase coherence (ITPC) and power are significantly higher for trough versus peak phase-locked sounds in the early (0–300 ms) and late (>300 ms) poststimulus time windows at both locations. ***C***, ***G***, Trough (blue lines) and peak (red lines) phase conditions generate opposing phase-dependent effects in early and late time windows for the baseline normalized alpha total power. ***C***, ***G***, Bottom, Permutation testing with post hoc cluster analyses finds total power is significantly higher for trough versus peak phase conditions in the early time for both locations. Only the frontal (Fpz, ***C***) location has significant phase-dependent differences in the later time window for total power. ***D***, ***H***, Alpha desynchronization or decrease in induced alpha power is larger for trough versus peak phase conditions in the later time window at frontal locations (***D***). ***D***, ***H***, Bottom, Permutation testing finds phase-dependent alpha desynchronization in the late time window is significant at frontal locations only.

**Figure 11.**
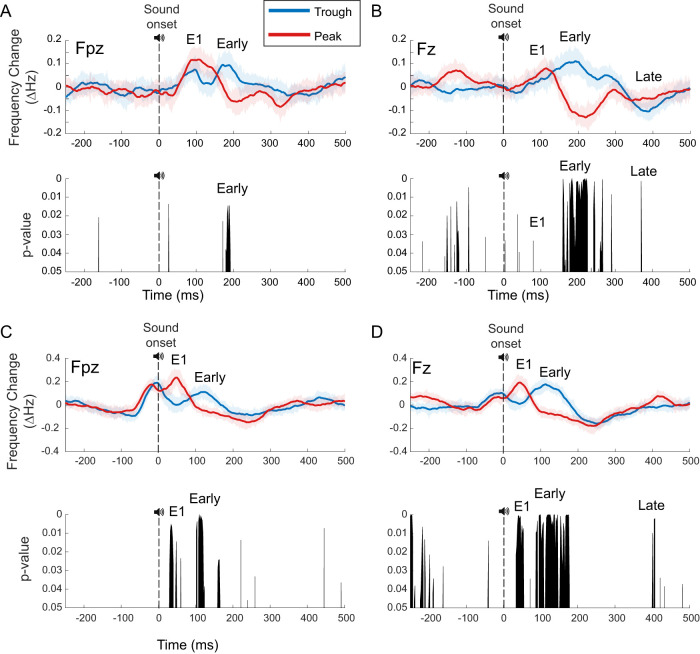
Phase-dependent change in evoked instantaneous alpha frequency across individualized trough and peak alpha phase conditions. ***A***, ***B***, Top, Standard Hilbert transform with causal filters used to analyze the instantaneous alpha frequency (Materials and Methods). With standard analysis, there are two apparent phase-dependent early responses labeled, E1 (<100 ms) and Early (<300 ms). ***A***, ***B***, Bottom, Permutation testing finds significant sustained alpha frequency changes with *p* value ≤0.04 during the Early time window (170–190 ms) at frontal (Fpz) location and the Early time window (160–225 ms) at the prefrontal (Fz) location. ***C***, ***D***, Top, ecHT with causal filters used to analyze the instantaneous alpha frequency (Materials and Methods). With ecHT analysis, there are two significant phase-dependent early responses labeled, E1 (<100 ms) and Early (<200 ms). ***C***, ***D***, Bottom, Permutation testing finds a continuous block of significant phase-dependent difference in frequency sliding with *p* values ≤0.01 in two time windows (E1 < 100 ms, Early <200 ms) at frontal (Fpz) location, and *p* values ≤0.0001 in the two time windows (E1 < 100 ms, Early <200 ms) at the prefrontal (Fz) location. There are additional less sustained alpha frequency changes indicated in the *p* value distributions. For both analyses, these phase-dependent changes in alpha frequency are more prominent at the prefrontal (Fz) versus frontal (Fpz) location. Exact *p* values indicated by *p* value distribution plots.

We visualized broadband (Figs. 8, 9) and quantified narrowband (Figs. 10, 11) evoked and induced responses to assess effects with higher and lower temporal coherence across trials, respectively (Helfrich et al., 2019). Evoked and induced responses were indexed here with intertrial phase clustering (ITPC), evoked power, total power, and induced power metrics, as described previously (David et al., 2006; Helfrich et al., 2019).

### ITPC

The ITPC assesses the temporal precision and cross-trial phase coherence of evoked responses. The auditory evoked ITPC was calculated as described previously (Tallon-Baudry et al., 1996; Cohen, 2014a). Instantaneous ITPC is treated as the absolute value of the averaged phase values across trials at each time point:

(5) $$\mathrm{ITPC}=\left|\frac{1}{N}\overset{N}{\underset{n=1}{\sum }}\;e^{i\theta_{i}}\;\right|,$$

where $\theta_{i}$ is the respective single-trial phase angle for the corresponding complex signal $\varphi_{i}$. For group-level statistics, subject-specific ITPC values were Fischer *z*-transformed before averaging and followed with nonparametric estimation of standard error.

The ITPC and evoked power assess the temporal precision of response phase and magnitude, respectively. The total power assesses the baseline normalized magnitude of the combined evoked and induced responses. Finally, the induced responses are estimated by subtracting the average evoked response from the total power, reflecting time-locked but nonphase-locked responses.

### Evoked alpha frequency change

To quantify phase-dependent differences in the evoked alpha frequency that were evident in broadband time-frequency spectrograms (Figs. 8*A*,*B*, 9*A*,*B*), we computed the instantaneous alpha frequency using an approach similar to those described previously (Cohen 2014b; Vázquez-Marrufo et al., 2019); however, here we employed the bandpass IAF (±25%) causal filter prior to applying the Hilbert transform to minimize smearing backward time. Following the Hilbert transform, the instantaneous frequency was calculated as the mathematical derivative of the phase of the EEG signal, which is then multiplied by the sampling rate over 2*π*. Additionally, the ecHT is known to significantly attenuate the Gibbs phenomena typically introduced during bandpass filtering (Schreglmann et al., 2021; Hebron et al., 2022; Bressler et al., 2023). Therefore, we corroborate instantaneous frequency analyses using causal plateau filters with an implementation via the ecHT.

### Task differences in prestimulus alpha power

Prior studies found that prestimulus alpha power levels must be high in order to see average or phase-dependent effects on the sensory ERP and alpha oscillations (Mathewson et al. 2011; Iemi et al., 2019). Hence, we aimed to confirm that our “trough and peak phase task” which used slow sound stimulation rates (0.611 Hz) resulted in high prestimulus alpha power levels, as observed in prior studies (Henry and Obleser 2012; Iemi et al., 2019; Cabral-Calderin and Henry, 2022). Here we examined the power (Fig. 4) in the prestimulus window using two methods and two time windows. For the first approach (Fig. 4*A–D*), we calculated prestimulus PSD using the FOOOF method during the prestimulus window (Iemi et al., 2019; Donoghue et al., 2020). Specifically, we confirm high alpha power during two, prestimulus windows of −0.5 s and −0.7 s (−500 ms and −700 ms) for the trough and peak phase conditions at frontal (Fig. 4*A*,*B*) and prefrontal (Fig. 4*B*,*D*) locations. For the second approach (Fig. 4*E*,*F*), we estimated the evoked power spectrum using a second approach (Cabral-Calderin and Henry, 2022). In this approach, the fast Fourier transform is taken of the average evoked activity across trials for each participant–electrode–condition interaction. When prestimulus activity is averaged before performing the FFT, oscillatory activity that is not time locked or phase locked to the stimulus onset in the prestimulus time window is averaged out. Hence, this procedure highlights signals which maintain consistent trial-by-trial time and phase alignment to sound onset. With the first approach, we observed high power levels in the alpha frequency range at both locations (Fig. 4*A–D*), and with the second approach, we observe high power levels in the delta and alpha frequency ranges at both locations (Fig. 4*E*,*F*). We used these same signal processing methods to quantify delta and alpha power prior to sound onset in the “random phase eyes open task” (Fig. 4*G–L*). In this task, we found low prestimulus alpha power levels as intended to measure auditory evoked potential latencies and amplitudes independent of alpha. Notably, we did not see high levels of phase coherence (ITPC) for delta oscillations in the prestimulus time window, as suggested in prior studies using different forms of analyses (Henry and Obleser 2012; Zoefel and Heil, 2013); however, we do see high delta power (Fig. 4*E–L*).

### Total and evoked power

As outlined in previous literature, total power quantifies the combined phase-locked and nonphase-locked activity, whereas evoked power quantifies only the phase-locked activity (David et al., 2006). Broad and narrow alpha band analyses were run using the same approach (Figs. 8–10, respectively). After calculation, the baseline power in the prestimulus period (−250 −50 ms) was subtracted from the total power (aka baseline normalized). Total power was quantified for each subject by taking the average magnitude squared across all trial-time-frequency points as follows:

(6) $$\mathrm{Total}\;\mathrm{Power}=\;\frac{1}{n}\overset{n}{\underset{i=0}{\sum }}\;|\varphi_{i}{|}^{2},$$

where $\varphi_{i}$ is taken as the instantaneous trial-time-frequency point. Similarly, evoked power is taken as the corresponding power to the averaged signal as follows:

(7) $$\mathrm{Evoked}\;\mathrm{Power}={\left|\frac{1}{n}\overset{n}{\underset{i=0}{\sum }}\;\varphi_{i}\right|}^{2}.$$

### Induced power

Induced power was quantified to estimate transient, time-locked but nonphase-locked activity. Unlike the evoked power or total power, to compute the induced power, we subtracted the ERP. Negative and positive changes in induced power, as calculated here, have been referred to as event-related desynchronization and event-related synchronization, respectively (Iemi et al., 2019). Here, we defined induced power as the average magnitude squared, frequency-specific activity after subtracting each subject's evoked activity from each trial and subsequently performing the complex Morlet wavelet transform as described above $\left(\varphi_{\hat{\text{ı}}}\right)$:

(8) $$\mathrm{Induced}\;\mathrm{Power}=\;\frac{1}{n}\overset{n}{\underset{\hat{\text{ı}}=0}{\sum }}\;|(\varphi_{\hat{\text{ı}}}){|}^{2}.$$

The alpha band delimited induced power (Fig. 10) was derived identically to the broadband metric of induced power but within the IAF band used throughout the study. After calculation, the baseline power in the prestimulus period (−250 to −50 ms) was subtracted from the induced power (aka baseline normalized) to allow direct comparisons to the total power.

### Statistical procedures

Nonparametric permutation testing was carried out to determine statistical differences in magnitudes of the ERP components, alpha ERO envelopes, alpha ITPC, evoked power, total power, and induced power (Maris and Oostenveld 2007; Cohen, 2014a). In brief, subject-specific trial-condition averages were taken. After which, for one thousand iterations, condition mean labels are swapped randomly, and the group average for the shuffled conditions were calculated. We then *z*-score our true mean differences relative to the resulting distribution and further estimate our permutation *p* values for each time point. Cluster-based correction is performed by taking from each permutation iteration the largest cluster of test statistics above our voxel significance threshold (*p* < 0.05). From which, our corrected cluster threshold is taken as the 95th percentile of the maximum cluster sizes over each permutation, and significant clusters are taken as those above said threshold. Unless otherwise noted, all variances reported in parentheses are the standard error of the mean (SEM).

For parametric analyses of ERP component latencies and amplitudes, two procedures were used. First, a multiway analysis of variance (MANOVA) was performed to investigate fixed effects of condition, electrode, and component on ERP component latency and amplitude. Given that the optimal phase of stimulation for both trough and peak phase conditions was estimated using P1 latency as determined by random-phase acoustic stimulation, we ran a MANOVA to examine the five component latencies across random, trough, and peak conditions. Second, in addition to permutation testing, we delineated trough versus peak phase-dependent differences in the five ERP component latencies and amplitudes and ran paired *t* tests followed by a Benjamini–Hochberg correction for multiple comparisons. Throughout the manuscript, standard deviations are reported in parenthesis.

Finally, statistical comparisons of circular data are performed primarily with two tests: Rayleigh's and Watson's *U*2. Rayleigh's test assesses the significance of nonuniformity of circular data, whereas Watsons *U*2 evaluates the significance of differences between two samples of circular data (Mégevand, 2017; Philipp Berens, 2024).

### Data and code availability

All code for all analyses are available here: https://github.com/readlabuser/Individualized-closed-loop-sensory-stimulation-reveals-alpha-phase-dependence.

## Results

### Estimating individualized target alpha phases

Based on known physiology, we hypothesize that sound evoked and induced effects will vary with the individualized alpha phase for sound onsets. Based on observations in the visual system (Alexander et al., 2020), we play sounds phase locked to trough and peak alpha phases that allow the sound evoked P1 potential to overlap with the following peak and trough alpha phases, respectively. To do this, we measure each subject's baseline IAF and their individual auditory ERP latencies using three different task conditions (Materials and Methods; Fig. 1). To determine IAF for each subject, we record alpha oscillations during the “baseline eyes closed task” (Fig. 1*Ai*,*Aii*). Time-frequency and PSD plots illustrate how the alpha frequency with maximum power is determined (Fig. 2). The population average spectrogram yields IAFs of 9.6 Hz at both recording locations (Fig. 2*C*,*D*, Fpz, Fz). The average of all the individually derived IAF measures for the entire group also is close to 10 Hz at both recording locations [*n* = 19 subjects, 10.32 (1.09) and 10.13 (0.85), Fpz and Fz, respectively]. Next, the “random phase eyes open task” is used to minimize alpha power as detailed below and to record auditory evoked responses to brief audible pink-noise stimuli presented at random alpha phases (Fig. 1*Bi*) to obtain an initial estimate of the P1 latency (Fig. 1*Bii*). Based on the individualized alpha and this P1 latency measured at the frontal location, we calculate the individualized trough and peak sound onset phases that allow the P1 component to coincide with the estimated following peak or trough phases of alpha, respectively (Fig. 1*D*,*E*). The auditory evoked P1 potential is used to estimate the individualized phase for each subject because all subjects have a prominent P1 potential (Fig. 1*Bii*,*Cii*), and this potential can be measured at frontal and prefrontal and central locations (Knight et al., 1999; Eggermont and Ponton, 2002). Using this approach, we compute the individualized target phases for sound onset based on the IAF and the P1 latency for each participant (Figs. 1*D*,*E*, 3, Fpz). In principle, a 10 Hz alpha frequency has an alpha cycle duration of 100 ms corresponding to a full cycle (360°) on a radial plot (Fig. 3). A hypothetical individual who has an IAF of 10 Hz and a P1 latency of exactly 50 ms would have an individualized trough phase for sound onset at 180° (Fig. 3*A*,*B*, pink dot) to align the P1 to the following maximum peak phase (Fig. 3*B*, 0°/360°). In contrast, the example subject with an IAF near 12 Hz and a P1 latency near 70 ms has a “trough” sound onset phase at 66° to align the P1 to the following peak phase (Fig. 3*A*,*B*, orange dot, 60°). Accordingly, the targeted sound onset phase varies with the P1 latency and IAF for each subject (Fig. 3*A*). Radial plots of the target trough and peak onset phases illustrate the range of target phases estimated to align the P1 to the following alpha phase for all subjects (Fig. 3*B*,*C*). Next, we assess the potential phase dependence of physiological responses while subjects perform the “Trough and Peak Phase Task,” with eyes closed (Materias and Methods; Fig. 1*C*). Physiological response metrics include the amplitudes and latencies of five sequential ERP components obtained with the random, trough, and peak phase conditions (Figs. 5, 6; Table 1). Radial plots illustrate that the targeted (Fig. 3*B*,*C*) and actual (Fig. 3*D*,*E*) trough and peak alpha phases for phase-locked sound onset are not significantly different at the frontal location (Watsons *U*2 test, trough *U* = 0.002, *p* = 0.350; peak *U* = 0.005, *p* = 0.300). A small but significant difference in the alpha phase at frontal (Fpz) versus prefrontal (Fz) recording locations is observed for both trough and peak phase-locked sounds (Watsons *U*2 test, trough *U* = 0.841, *p* = 0.044; peak *U* = 0.817, *p* = 0.045). The P1 component latency averaged across all randomized phases and subjects is 64 ms (8 ms) and 57 ms (5 ms) for the frontal (Fpz) and prefrontal (Fz) locations, respectively (Table 1; *n* = 19). Within subject comparisons find minimal (<2 ms) mean P1 latency differences across random, trough, or peak phase-locked sound conditions at frontal (Fpz) locations (see Materials and Methods). Next, we confirm that the alpha phases immediately preceding and following trough versus peak phase-locked sounds are stationary, as indicated by their high correlation when sounds are played phase locked to trough (*r* = 0.99 ± 0.02) or peak (*r* = 0.99) alpha phase (Materials and Methods). Given this initial phase stationarity, the P1 potentials evoked by trough and peak phase-locked sounds occur on average during the following peak and trough phases, respectively, at frontal (Fig. 3*F*,*G*, Fpz) and prefrontal (Fig. 3*H*,*I*) locations. Therefore, the mean trough and peak evoked P1 potentials fall on opposite halves of the alpha cycle, as intended. Though sounds are phase locked to frontal (Fpz) alpha, the sound evoked P1 potentials coincide with similar alpha phases at frontal (Fig. 3*F*,*G*) and prefrontal (Fig. 3*H*,*I*) locations and the corresponding phases are not significantly different (Materials and Methods; trough *U*2 = 0.065, *p* = 0.559; peak *U*2 = 0.093, *p* = 0.321).

### Task-dependent high prestimulus alpha levels

Prior studies found that high levels of alpha are necessary to generate a variety of stimulus evoked and induced effects including changes in alpha oscillations and evoked potentials (Mathewson et al., 2009; Iemi et al., 2019; Dou et al., 2022). Here, the “random phase eyes open task” is designed to reduce prestimulus alpha levels to obtain stable measures of the P1 latency independent of alpha. In contrast, the “trough and peak phase task” is designed to promote high prestimulus alpha levels (Materials and Methods). Accordingly, in the “trough and peak phase task,” subjects are instructed to ignore the sounds and to rehearse a multiplication table with eyes closed while phase-locked sounds are played with a long ISI [ISI = 1,636 (55) ms] and corresponding slow (0.611 Hz) stimulus rate (Fig. 1*C*,*Ci*). To assess alpha levels, we quantify the normalized broadband PSD in two prestimulus time windows (Fig. 4). In the 500 ms time window prestimulus, we found alpha levels are high in the “trough and peak phase task” (Fig. 4*A*,*B*). These levels are comparable with high alpha levels associated with open-loop visual (Mathewson et al. 2011; Iemi et al., 2019) and auditory (Henry and Obleser 2012; Cabral-Calderin and Henry, 2022) stimulus evoked and induced effects. When the same analysis is performed for the 700 ms time window prestimulus (Fig. 4*C*,*D*), the maximal alpha power level is reduced suggesting that alpha levels rise in the 500 ms prior to stimulation on average. Using a second signal processing approach that highlights alpha that is time locked and phase locked to stimulus onset (Materials and Methods; Cabral-Calderin and Henry, 2022), we found prestimulus delta and alpha power are both high in the “trough and peak phase task” condition (Fig. 4*E*,*F*). Permutation testing finds significant differences in the beta and gamma frequency ranges (Fig. 4*A*,*B*,*C*, gray box overlay) for trough versus peak phase-locked conditions. Importantly, there are no significant differences in prestimulus delta or alpha power across trough versus peak phase conditions in this task (Fig. 4*A–F*). In contrast, during the “random phase eyes open task,” the prestimulus alpha levels are low with both alpha power analyses (Fig. 4*H–J*) and delta levels are high with the second analysis method (Fig. 4*I*,*L*). Thus, the “random phase eyes open task” allows for measures of the auditory ERP independent of alpha oscillations, and the “trough and peak phase task” allows for high prestimulus alpha levels that have been shown to be necessary for phase-dependent and phase-independent sensory stimulus-induced effects.

### Accuracy of close-loop individualized alpha phase-locking

This study uses a novel ecHT algorithm implemented on a research device that records EEG to play sounds accurately phase locked to instantaneous individualized alpha phase. As illustrated with radial phase plots, target (Fig. 3*B*,*C*) and actual (Fig. 3*D*,*E*) phases cover a similar mean and total range across individualized trough and peak phase conditions. Next, the trial-by-trial probability that the actual phase matches the target phase is quantified with the PLV metric and the phase-targeting precision is quantified with the PE metric. Both metrics are calculated for each subject individually and averaged across trough and peak phases, which were not significantly different in PLV (*t*_(18)_ = 1.03; *p* = 0.316). The mean PLV is high when phase is estimated with the endpoint corrected ecHT algorithm coupled with a causal filter, as implemented on the device (Fig. 3*J*; PLV = 0.92 ± 0.03). This indicates a 92% probability of hitting the targeted phase across all sound trials with the ecHT approach. The average PE between target and actual phase is under 10° phase angle with the ecHT (Fig. 3*J*; PE = −9° ± 5°). When these metrics are computed with the standard Hilbert transform using a causal filter, the mean PE is higher and the mean PLV is lower but still above chance levels (Fig. 3*K*). These results confirm prior studies finding this ecHT technology generates accurate real-time biological and EEG-based phase-locking (Schreglmann et al., 2021; Hebron et al., 2022; Bressler et al., 2023). Here, we extend this prior work by demonstrating high phase-locking accuracy when using slow sound stimulation rates with individualized target alpha phases. This high degree of phase-locking accuracy allows us to examine the alpha phase-dependent effects on physiological responses following sound onset.

### Auditory evoked potentials vary with alpha phase at stimulus onset

Multiple studies have found that early sensory evoked responses vary with the alpha phase coinciding with visual (Fellinger et al., 2011; Haegens and Golumbic 2018; Dou et al., 2022) or auditory stimulation (Kruglikov and Schiff, 2003). To our knowledge, there are no prior studies examining how auditory evoked responses vary with the alpha phase measured at frontal locations. As detailed above, we confirm within subject consistency (<2 ms) of the P1 latency during the random versus trough and peak phase-locking tasks. Using appropriate time windows, we compare amplitudes (Figs. 5*C*,*D*, 6*C*,*D*) and latencies (Fig. 6*A*,*B*) of five auditory ERP components across random, trough, and peak phase-locked conditions. Permutation testing and *t* tests are used to assess significant changes in component amplitudes and latencies with phase condition (Materials and Methods). No significant differences in the five ERP component latencies are found across trough, peak, and random phase conditions supporting the concept that the P1 potential is aligned to predicted poststimulus alpha phases in both task conditions (trough, peak, random MANOVA: *F*_(2,72_) = 2.05, *p* = 0.130; trough versus peak *t* test; Table 1). When comparing ERP component amplitudes, we found the early positive (Pa) component of the ERP is ∼1.6-fold smaller for trough versus peak conditions at both locations (Fig. 5*C*,*D*, see “Pa” components). Accordingly, permutation tests find the Pa potential is significantly smaller for trough versus peak phase conditions (Fig. 5*C*,*D*, see shaded area labeled “Pa” in *p* value distribution). In contrast, permutation testing finds three subsequent ERP components (P1, N1, P2) have significantly larger amplitudes with trough versus peak phase conditions at both locations (Fig. 5*C*,*D*). These phase-dependent effects on the Pa, P1, and N1 component amplitudes observed with nonparametric permutation are confirmed by secondary *t* test analyses (Table 1, bottom panels). Given that early prestimulus alpha power does not significantly vary across trough versus peak phase conditions (Fig. 4), these phase-dependent differences in Pa, P1, and N1 amplitudes are not likely due to a difference in the prestimulus alpha power which is known to impact ERP amplitudes (Iemi et al., 2019). As in the current study, trough and peak phase-locked sounds have opposite effects on the auditory evoked Pa versus P1 and N1 potentials measured at more central (Cz) locations (Kruglikov and Schiff, 2003). However, here we found the phase polarity is reversed for these effects with frontal recording locations. These alpha phase-dependent effects on auditory evoked potential amplitudes are consistent with the current theory that alpha provides a phasic or gating inhibition of sensory processing (Bonnefond et al., 2017). Though this study measures the auditory ERP and alpha phases at frontal locations, additional studies with a large EEG electrode array are needed to confirm the actual brain sources that may underlie the phase-dependent effects.

### Alpha band-delimited EROs vary in magnitude with individualized alpha phase

Next, we confirm that the average alpha band-delimited ERO is aligned to the target phases and then compare the phase-dependent changes in this narrowband signal following sound onset (Materials and Methods). Though all sound phase-locking is targeting frontal (Fpz) alpha phase, the mean alpha ERO phase polarity for trough and peak sound onset is similar at both recording locations (Fig. 7). At stimulus onset time, the mean ERO phase is aligned to the alpha oscillation trough (blue lines) and peak (red lines) phases, as intended for these respective stimulus conditions (Fig. 7*A–D*). For both alpha phases and locations, the alpha oscillation variance is markedly reduced immediately poststimulation (Fig. 7*A–D*, see decrease in shaded areas). For the trough phase condition, alpha oscillation amplitude remains high initially following stimulus onset, at both locations (Fig. 7*A*,*B*). With the peak phase condition, the ERO amplitude decreases in the early time window following stimulus onset at both locations (Fig. 7*C*,*D*). Permutation tests find the evoked alpha envelope magnitude is reduced more so for the peak versus trough phase conditions in the early time window (<300 ms), at both locations (Fig. 7*E*,*F*, bottom *p* value distribution). This difference in evoked alpha envelope magnitude between trough and peak phase conditions is greater at the more posterior prefrontal (Fz) location (Fig. 7*G*,*H*). Overall, the poststimulus alpha magnitude is larger at the prefrontal versus frontal EEG recording locations (Fig. 7*I*,*J*). In theory, the posterior prefrontal (Fz) location may reflect source activity from auditory cortical activity on Heschl's gyrus, as summarized across multiple studies (Eggermont and Ponton, 2002). Independent of the source localization, these results support the hypothesis that sound evoked neuromodulation of alpha oscillations varies with the individualized phase of alpha.

### Alpha phase dependence of auditory evoked and induced alpha oscillations

Sensory events can “evoke” and “induce” EEG responses across repeated stimulation with high and low degrees of synchrony, respectively (David et al., 2006; Helfrich et al., 2019). Here, we assess phase-dependent changes in sound evoked and induced EEG responses by computing the ITPC, instantaneous frequency change, and evoked, total, and induced power across broad (Figs. 8, 9) and narrow (Figs. 10, 11) spectral frequency ranges (Materials and Methods).

Real-time phase-locked sound presentation reveals an alpha phase dependence for multiple evoked brain response metrics including alpha phase coherence, alpha power, and instantaneous alpha frequency. First, for both trough and peak phase-locked sounds, the average ITPC and evoked alpha power decrease following sound onset, as evident in standard broadband (Figs. 8, 9*A*,*B*) and alpha narrowband (Fig. 10*A*,*B*,*E*,*F*) time-frequency analyses. This phase-independent effect likely emerges due to our use of salient, loud sounds with maximum sound levels of 85 decibels, based on prior studies (Peng et al., 2012; Fodor et al., 2020). Second, permutation testing finds the narrowband evoked alpha ITPC and alpha power are significantly higher for trough (blue) versus peak (red) phase conditions in the early (<300 ms) and later (>300 ms) time windows poststimulation at both locations (Fig. 10*A*,*B*,*E*,*F*, see “early” and “late” *p* value distributions). This is consistent with the high poststimulation alpha ERO amplitude following trough phase-locked sounds (Fig. 7*A*,*B*) and resembles the alpha phase dependence of ITPC observed with post hoc analyses of alpha following visual stimulation aligning the early (C1) visual evoked potential to poststimulus alpha phase (Alexander et al., 2020). Third, we found distinct instantaneous alpha frequency changes for trough versus peak phase-locked conditions as measured with standard and endpoint corrected Hilbert transformed data using causal filters (Materials and Methods; Fig. 11). With the ecHT metric, in the first 100 ms following sound onset, alpha frequency decreases and increases for trough versus peak phase conditions, respectively (Fig. 11*C*,*D*, blue vs red lines, labeled E1). With both metrics, in the 100–300 ms time window following sound onset, the alpha frequency increases and decreases for trough versus peak phase conditions, respectively (Fig. 11*A–D*, see “Early”). The alpha frequency differences across phase conditions are larger in magnitude (∼0.3 vs ∼0.2 Hz) and occur over shorter time scales (<200 vs <300 ms) for the ecHT versus standard Hilbert transform analyses. Permutation testing finds these opposing phase-dependent changes in instantaneous alpha frequency are significant for both metrics and recording locations (Fig. 11, *p* value distributions) but are more prominent and sustained at the prefrontal (Fz) location (Fig. 11*B*,*D* vs *A*,*C*). Similarly, there are phase-dependent changes in alpha frequency in the 100–300 ms time window following sound onset evident in the broad-spectrum plots particularly for the prefrontal electrode (Figs. 8*E*,*F*, 9*E*,*F* see frequency change in ITPC and power between white dotted lines). Importantly, these changes in alpha power and phase coherence follow a high degree of alpha phase coherence prior to stimulus onset as shown with the broadband time-frequency power plots (Figs. 8, 9*A–F*, prestimulus, red area). Collectively, these alpha phase-dependent effects on evoked alpha ITPC, alpha power, and alpha frequency are consistent with the idea that sound-driven neuromodulation of alpha varies with the alpha phase at the time of sound onset. Moreover, these results extend prior work by demonstrating how sounds phase locked to individualized frontal trough versus peak alpha phase evoke distinct changes in instantaneous alpha oscillation frequency.

Finally, we question whether there are phase-dependent induced effects on alpha. As the sound evoked ERP is removed to quantify the induced effects, this metric examines effects on alpha oscillations that are not time locked to the stimulus onset (Materials and Methods). First, we quantify both the total and “induced” changes in alpha power normalized to the prestimulus baseline alpha power to index stimulus-dependent changes. With broadband frequency analysis, trough phase-locked sounds cause an early increase followed by a later decrease in the baseline corrected total alpha power (Figs. 8*C*, 9*C*, arrow). In contrast, peak phase-locked sounds cause small baseline corrected decrease in total alpha power (Figs. 8*G*, 9*G*, arrow). The narrowband total alpha power increases and decreases relative to baseline (dotted line) for trough versus peak phase conditions in the early time window (<300 ms) following sound onset at both locations (Fig. 10*C*,*G*). In contrast, the narrowband total alpha power decreases and increases relative to baseline (dotted line) for trough versus peak phase conditions in the late time window (>300 ms) following sound onset at both locations (Fig. 10*C*,*G*). Permutation testing finds significant phase-dependent differences in the alpha total power corresponding to the early (<300 ms) time window at both recording locations (Fig. 10*C*,*G*, Early). Significant phase-dependent differences are observed in the late time window at the frontal (Fpz) location only (Fig. 10*C*, Late). In the early time window prior to 300 ms, no differences are observed for the induced alpha desynchronization at either recording location (Fig. 10*D*,*H*). In the later time window (>300 ms), permutation tests find a significant decrease in alpha level for trough versus peak phase conditions at the frontal (Fpz) location only (Fig. 10*D*,*H*). Thus, the biphasic increase and decrease in baseline corrected total alpha power with the trough phase could reflect an increase in evoked alpha power in the early time window followed by a decrease in induced alpha power in the later time window. As prior work finds the magnitude of alpha desynchronization scales with the preceding alpha levels (Iemi et al., 2019), the larger degree of alpha desynchronization for trough versus peak phase conditions likely reflects the higher alpha levels in the early time window poststimulation. Collectively, these results indicate distinct alpha phase-dependent effects of auditory events on evoked and induced responses over early and late time windows.

## Discussion

This study supports the feasibility to use a real-time, automated, closed-loop technology to record EEG and play sounds phase locked to alpha oscillations with a high degree of precision and low PE. Using this technology, we demonstrate dynamic phase-dependent interactions between sound onset, evoked responses, and alpha oscillations measured with EEG in humans. Additionally, we demonstrate feasibility to use individualized alpha and sensory ERP indices to play sounds phase locked to instantaneous alpha phase in real time. These results extend prior work by showing multiple evoked and induced effects when playing sounds at different alpha phases.

Several main results support the general concept of alpha phase-dependent neuromodulation in the auditory system. First, we found IAF and auditory evoked P1 potential latencies vary across subjects and use these parameters to calculate target trough and peak alpha phases that allow the P1 potential to occur during the subsequent peak and trough phases, respectively. Second, we found the alpha phases preceding and following phase-locked sound onsets are highly (*r* = 0.99 or 99%) correlated suggesting that the alpha phase is relatively stationary in the earliest time window following sound onset. Third, we found a low PE between target and actual phase for playing sounds. Fourth, we found that trough and peak phase-locked sounds evoke P1 potentials that occur close to one-half of an alpha cycle after sound onset, as intended with our individualized phase-locking approach (Fig. 3*F–I*). Fifth, we found the early auditory evoked Pa potential is lower and higher amplitude for trough versus peak phase-locked conditions, respectively. Prior work has shown higher Pa amplitudes with loss of the suppressive cortical feedback from the frontal lobe to auditory cortices (Knight et al., 1999). In theory, the increase in Pa potential amplitude could reflect a stimulus-induced decrease in frontal cortical feedback to auditory cortices with peak phase-locked sounds; however, additional studies are required as detailed below. In principle, this effect is consistent with alpha phase dependence of the early visual evoked thalamocortical potential (Dou et al., 2022). Our sixth finding is that the P1 and N1 components of the ERP are decreased in amplitude more so when sound is played at individualized alpha peak versus trough phase. The latter result suggests that P1 potential is more likely to be suppressed if it arrives during the presumed “excitable” trough phase of alpha that follows sound onset during the alpha peak phase. Though prior work has localized alpha oscillations to frontal sources, there appear to be contributions to alpha and other oscillations from spatially remote brain areas (Srinivasan et al., 2006). Thus, additional studies are needed to establish the brain sources contributing to this phase dependence and to determine causal relationships, as detailed below. A seventh result is a phase-dependence of the evoked alpha oscillations that follow phase-locked sounds, as observed in the visual system (Otero et al., 2020). Here, we extend prior work finding the evoked alpha ERO, ITPC, and power all decrease with the peak phase-locked sounds poststimulation. In contrast, with trough phase-locked sounds, the evoked alpha ERO, ITPC, and power all stay the same or increase at frontal (Fpz) and prefrontal (Fz) locations, respectively. An eighth and novel finding is alpha phase-dependent effects on instantaneous alpha frequency following trough versus peak phase-locked sounds, respectively. Notably, this effect is most prominent in the time windows overlapping with the P1, N1, and P2 components (Fig. 11). Prior studies found damage to the lateral prefrontal cortex alters auditory evoked potentials in this same time window when subjects participate in tasks requiring them to ignore distracting sounds (Bidet-Caulet et al., 2015). Hence, it will be of interest in the future to determine whether these phase-dependent changes in alpha frequency are correlated with attentional effects and causally related to lateral prefrontal cortical feedback to auditory cortices or alternative sources. Finally, we observe a phase-dependent difference in induced alpha desynchronization which mitigates potential contributions of the ERP signal to poststimulus alpha signals. For trough phase-locked sounds, the late induced alpha desynchronization follows a net increase in evoked alpha level in the early time window. This is consistent with prior studies finding that the capacity to induce alpha desynchronization scales with the alpha levels in preceding time windows (Iemi et al., 2019). Collectively, these results lend support to the theory that auditory evoked and induced brain responses vary in an alpha phase-dependent manner on multiple time scales.

### Closed-loop alpha phase-dependent modulations of evoked and induced responses

Few studies have used closed-loop technologies to play sounds phase locked to alpha oscillations to quantify phase-dependent effects. As reviewed previously, several studies fail to show alpha phase-dependent effects with a variety of open-loop rhythmic sound stimulation protocols and post hoc phase analyses alone (VanRullen et al., 2014; Zoefel and VanRullen, 2017). Conversely, with direct open-loop, rhythmic, transcranial current stimulation of the auditory cortex, alpha levels are elevated, and auditory signal detection rates vary with alpha phase (Neuling et al., 2012). Consistent with the later result, the auditory ERP measured at central (Cz) EEG locations varies with trough alpha phase detected with voltage thresholding versus following phases at fixed time intervals (Kruglikov and Schiff, 2003). Auditory evoked responses at central (Cz) locations are typically source localized to the primary and belt auditory cortices on Heschl's gyrus (Eggermont and Ponton, 2002). Here, we found an alpha phase-dependent enhancement of the auditory evoked Pa potential coupled with a suppression of the following P1 and N1 potentials for peak alpha phase-locking measured at frontal (Fpz) locations. Likewise, the phase-dependent effects on the auditory evoked Pa potential are opposite of those on the P1 and N1 potentials when sounds are phase locked to alpha measured at a central (Cz) location (Kruglikov and Schiff, 2003). However, the alpha phase-dependent effects shown here with frontal (Fpz) electrode recordings are reversed in phase polarity compared with those observed at central (Cz) locations. These response and phase polarity differences across studies could reflect known shifts in alpha phase polarity between frontal and more posterior EEG or intracranial recording locations (Bahramisharif et al., 2013; Halgren et al., 2019; Alamia et al., 2020; Pang et al., 2020). Supporting this possibility, we observe small but significant differences in the alpha phase at the time of sound onsets for frontal (Fpz) versus prefrontal (Fz) recording locations. In addition, task-related engagement of different cortical brain areas could in theory impact the polarity of the phase-dependent effects of sound onsets. Finally, differences in the temporal alignment of the auditory evoked P1 potential with a given alpha phase could in theory impact these phase-dependent effects. Though supportive of our hypotheses, the overlap between a given alpha phase and the auditory P1 potential may not causally drive the phase-dependent effects we observe here. Future studies are necessary to clarify whether top-down cognitive processes influence these effects and what cortical sources drive the alpha phase dependence of sound evoked and induced physiological responses observed here.

### Need for future large-scale recordings with source localizations

Prior work supports the potential involvement of auditory and frontal cortical areas for the phase-dependent effects observed here but additional source localization is necessary to confirm the true sources. Across many mammals, frontal and higher level auditory cortical areas provide top-down regulation of auditory processing and attention through feedback projections (Knight et al., 1999; Medalla and Barbas, 2014; Plakke and Romanski 2014; Bidet-Caulet et al., 2015; Dimitrijevic et al., 2019). Frontal and auditory cortices generate alpha oscillations during passive and active listening and when actively ignoring sounds (Dubé et al., 2013;Mazaheri et al., 2014; Gray et al., 2015; Haegens et al., 2015; Zhang et al., 2018; Billig et al., 2019; Halgren et al., 2019; Wöstmann et al., 2019; Clements et al., 2022). Alpha can be localized to frontal cortices with large-scale EEG recordings (Srinivasan et al., 2006). Primate frontal and auditory cortices both have short latency auditory evoked responses, as demonstrated with intracranial recordings by our group (Marrouch et al., 2020) and others (Howard et al., 2000; Happel et al., 2010; Haegens et al., 2015; Komatsu et al., 2015; Kajikawa et al., 2017). However, with EEG source localization analyses, the auditory evoked Pa and P1 potentials measured in the present study are typically attributed to activation of primary and secondary auditory cortical areas, respectively (Näätänen and Winkler, 1999; Eggermont and Ponton, 2002; Itoh et al., 2021; Kohl et al., 2022). Additionally, alpha and other synchronous oscillations measured with EEG can have source contributions distributed over larger spatial scales (Srinivasan et al., 2006). Thus, the phase-dependent effects reported here between auditory evoked potentials and alpha oscillations may very well be due to physiological interactions in nonfrontal cortical areas including Heschl's gyrus. In an analogous fashion, evoked responses to open-loop visual stimulation vary with localized sources of alpha oscillatory activity (Nuttall et al., 2022). As signals are recorded at only two scalp locations in the present study, future studies providing EEG source localization are necessary to localize the underlying brain sources of alpha and auditory evoked potentials giving rise to the phase-dependent effects observed here.

### A need for perceptual and behavioral measures

The current study aims to determine feasibility of using closed-loop phase-locking to determine if evoked and induced physiological responses vary with individualized alpha phase at sound onset. However, it will strengthen our understanding of these phase-dependent effects if future studies include trial-by-trial measures of neurophysiological and task performance metrics. For example, when people attend to visual events while ignoring auditory events, the trial-by-trial alpha level and reaction times are significantly correlated in occipital, central, and frontal cortical locations (Mazaheri et al., 2014). The latter suggests that high alpha levels in occipital, central, and frontal cortical locations are necessary to effectively ignore distracting auditory stimuli in order to respond to visual stimuli with faster reaction times. Conversely, when people attend to auditory stimuli while ignoring visual stimuli, the trial-by-trial correlations of alpha level and reaction times are highest within a central parietal cortical area suggesting that alpha in these areas helps people effectively ignore the visual input to respond more quickly to auditory stimuli (Mazaheri et al., 2014). Having such trial-by-trial correlative indices also could help distinguish response versus stimulus related phase-dependent effects (Makeig et al., 1999). Moreover, these metrics could help delineate whether reaction times are reliably faster when auditory events coincide with a particular phase of alpha, as observed in the visual system (Dustman and Beck 1965). Such metrics would help delineate whether the auditory system is perceptually “decoupled” from external auditory input when alpha levels are high (Zoefel and VanRullen, 2017) or alternatively whether auditory evoked brain and perceptual responses vary dynamically with instantaneous alpha phase.

### Feasibility to develop individualized and automated closed-loop stimulation approaches

In general, this study strengthens the feasibility to develop individualized and automated closed-loop stimulation approaches to explore new ways to modulate synchronous oscillations for research and health applications. Identification of individual neurophysiological indices is key to developing optimal protocols for intracranial and EEG-based closed-loop phase-locked or neural feedback remediation (Buzsáki and Watson, 2012; Mansouri et al., 2018; Sullivan et al., 2021; Widge 2023). For example, individuals with epilepsy, attentional deficit hyperactivity disorder, hyperarousal sleep disorders, tinnitus, Alzheimer's, and Lewy body dementias all have altered individualized alpha oscillation power, frequency, or variance (De Ridder et al., 2007; Weisz et al., 2011; Riedner et al., 2016; Caspary and Llano, 2017; Vanneste et al., 2018; Abela et al., 2019; Deiber et al., 2020; Ranasinghe et al., 2020; Schumacher et al., 2020; Yaakub et al., 2020; Schoisswohl et al., 2021; Zhao et al., 2021). Similarly, essential tremors are accompanied by altered synchronous oscillations in the theta frequency range in motor thalamus (Kane et al., 2009), and closed-loop deep brain stimulation can counteract these tremors (He et al., 2021). Accordingly, the ecHT device used in the present study has been used to successfully track essential tremor oscillations and deliver phase-locked transcranial direct stimulation to effectively reduce tremors (Schreglmann et al., 2021). Given that alpha levels are elevated in people with hyperarousal insomnia, we and others have used this ecHT technology to phase-lock to alpha measured with EEG at frontal (Fpz; Bressler et al., 2023), prefrontal (Fz) and parietal (Pz) locations (Hebron et al., 2022) locations to examine the potential to reduce hyperarousal and promote healthy sleep onset. The current study finds similarly high phase-locking accuracy to prior studies using the ecHT technology (Hebron et al., 2022; Bressler et al., 2023). Unlike prior studies, the current study employs a task that imposes a cognitive load to promote subjects to stay awake and ignore the sounds. Additionally, sounds are played infrequently with long ISIs instead of being played phase locked to sequential 10 Hz alpha oscillations separated by as little as 100 ms. With slow (0.611 Hz) or faster (10 Hz) stimulation rates, the PE remains low and well within the range needed to target peak or trough phases of alpha on average (Schreglmann et al., 2021; Hebron et al., 2022; Bressler et al., 2023). Though the present study achieves accurate phase-locking at slow rates (0.611 Hz) using individualized target phases based on IAF and ERP latencies, Hebron and colleagues found a high correlation between IAF and the actual ISIs achieved when sounds are played with continuous phase-locking to sequential nonindividualized alpha oscillations frequencies (Hebron et al., 2022). The latter indicates that the ecHT technology has the capacity to phase-lock to individual alpha frequencies under various automated phase-locking conditions. Previously, we found the ecHT phase-locking error is reduced by including more trials and subjects (Bressler et al., 2023). Additionally, simulations suggest that phase-locking accuracy could be further optimized by modifying the ecHT filter bandwidth to fit the natural exponential distribution of synchronous oscillation frequencies (Bressler et al., 2023). Collectively, these findings strengthen feasibility to develop more individualized closed-loop stimulation approaches to optimize dynamic neuromodulation of intrinsic brain activity with a variety of stimulus delivery systems.

## Synthesis

Reviewing Editor: Silvia Pagliardini, University of Alberta Decisions are customarily a result of the Reviewing Editor and the peer reviewers coming together and discussing their recommendations until a consensus is reached. When revisions are invited, a fact-based synthesis statement explaining their decision and outlining what is needed to prepare a revision will be listed below. The following reviewer(s) agreed to reveal their identity: Biao Han. Note: If this manuscript was transferred from JNeurosci and a decision was made to accept the manuscript without peer review, a brief statement to this effect will instead be what is listed below.

The manuscript investigates the alpha phase-dependence of auditory evoked responses using scalp electroencephalogram (EEG) recordings. The use of a novel end-point corrected Hilbert transform (ecHT) algorithm for real-time tracking of alpha phases during auditory stimulation is a noteworthy methodological innovation. The study aims to demonstrate the feasibility of closed-loop, phase-locked auditory stimulation. While the manuscript presents interesting findings, there are several points that require clarification and improvement.

The authors improved the manuscript and replied to previous points. Overall, I do find the research question interesting and methodology exciting for potential translational research in the future. Nevertheless, I have some remaining comments and questions which I describe below.

Major

1. While I appreciate the authors' effort to motivate the study of alpha phase-dependence in the introduction, the motivation to use closed-loop auditory stimulation is still lacking.

2. I'm also still missing an explanation why the authors expect to find alpha phase effects when participants are instructed to *ignore* sounds. I agree that alpha power can increase for ignored stimuli. However, this seems to conflict with the assumption that the phase of alpha modulates stimulus-evoked responses. How can a process that serves to *suppress* a stimulus (i.e. alpha power increases for ignored stimuli) be important for the *processing* of that stimulus at the same time?

3. I don't think the previous point "that a stimulus-induced phase-reset (that is often assumed) would de-correlate pre- and post-stimulus phases" is much a "detail" as the authors claim. The notion that stimulus processing is different when the pre-stimulus peak (had it continued) coincides with the P1 peak (as compared to when it does not) is central for this study yet not very common in the field. I believe that more efforts are required to justify this assumption. For instance:

- A (1) measure of correlation between pre- and post-stimulus phases, and (2) a comparison of the obtained results with a version that does not include post-stimulus data for the estimation of pre-stimulus phase would be useful.

- The authors used the latency of the P1 component to estimate "best" and "worst" phases at sound onset. It remains unclear what the advantage is of using the P1 latency over simply testing which alpha phase leads to the largest P1 amplitude. With only two data points (trough and peak phases), the argument that the two phases represent highest and lowest excitability brain states, respectively, is assumed but not verified. Another potential caveat of this approach is the consistency in P1 latency. Trials in which this latency deviates from the average might lead to an imprecise phase estimate. A measure of P1 reliability would be useful.

4. The ecHT method seems to be of critical importance in the current study, however, there is no explanation of how the ecHT works in the manuscript. As it is a rather novel method, I recommend to provide additional details on the algorithm behind ecHT and how it is advantageous compared to sHT.

5. In general, I believe the interpretation of the current results, which relies on only two scalp electrodes, is too speculative. Previous studies have demonstrated that while there is a contrast in excitability in the peak and the trough phase of LFP data, the phase effects are a lot more complicated on the scalp level. The absence of perceptual measures prevents the authors from drawing conclusions on the perceptual relevance of the phasic modulation and peak/trough phases, and this should be discussed. Also, the authors still put a lot of emphasis on the thalamocortical network in explaining the observed phase-dependence, which for me is highly speculative.

6.Frontal Electrode Selection:

The manuscript falls short in elucidating the rationale behind the choice of frontal electrodes (Fz and Fpz) for investigating alpha phase-dependence during ignored auditory stimulation. Frontal electrodes are conventionally linked to attention or task-related processes, and their direct relevance to the auditory system requires clarification. The limited presentation of data from a comprehensive array of electrodes diminishes the overall credibility of the results. A more robust approach would involve showcasing results from all electrodes and providing a well-founded justification for the specific selection of frontal electrodes within the auditory processing context.

7.Sensory-Driven vs. Top-Down Activity:

The claim that the results indicate an alpha phase dependence of sensory-driven brain activity requires further substantiation. The manuscript lacks evidence demonstrating that the responses observed in the frontal area are indeed the largest in response to auditory stimuli. Additionally, the choice of electrode layout may be suboptimal for capturing auditory responses, as central electrodes are conventionally used for auditory evoked potentials (AEP). The authors should either present data supporting the sensory-driven nature of the observed activity or modify their claim accordingly.

8. Inconsistency in Experimental Design:

What is the rationale behind using two different behavioural tasks (passive listening and mental arithmetic tasks) for the "random phase eyes open" and "peak and trough phase" tasks, respectively? Please explain.

The employment of eye-closed conditions for peak and trough phase tasks, coupled with eye-opened conditions for random phase tasks, introduces a notable inconsistency in the experimental design. This choice prompts queries about the underlying rationale and may potentially affect the interpretability of the results. Moreover, it is noteworthy that random phase tasks were incorporated in certain analyses, such as ERP Latency, but were omitted in others, like ERP amplitude analysis. To bolster the robustness and coherence of the study, the authors are encouraged to elucidate the reasoning behind this experimental design or contemplate aligning the conditions consistently across various analyses.

9. Post-Stimulus Alpha Activity:

The manuscript makes several claims about post-stimulus alpha activity without addressing potential contamination from event-related potentials (ERPs). The lack of decoupling between post-stimulus alpha and ERPs raises concerns about the scientific rigor of the claims. To enhance credibility, the authors should either provide evidence supporting the distinction between post-stimulus alpha activity and ERPs or consider removing claims related to post-stimulus results.

Minor comments

1. Did the participants receive compensation for taking part in the experiment?

2. I recommend to proofread and polish sentences for increased readability. There are still typos here and there (e.g., "pres-stimulus time period").
